# Assessment of synergy-assisted EMG-driven NMSK model for upper limb muscle activation prediction in cross-country sit-skiing double poling

**DOI:** 10.3389/fbioe.2025.1585127

**Published:** 2025-08-18

**Authors:** Xue Chen, Zhongxue Yuan, Xianzhi Gao, Yanxin Zhang, Chenglin Liu, Bo Huo

**Affiliations:** ^1^ School of Aerospace Engineering, Beijing Institute of Technology, Beijing, China; ^2^ Department of Exercise Sciences, The University of Auckland, Auckland, New Zealand; ^3^ Sport Biomechanics Center, Institute of Artificial Intelligence in Sports, Capital University of Physical Education and Sports, Beijing, China; ^4^ Emerging Interdisciplinary Platform for Medicine and Engineering in Sports (EIPMES), Beijing Municipal Education Commission, Beijing, China

**Keywords:** EMG-driven model, muscle synergy, cross-country sit-skiing, muscle activation, upper limb

## Abstract

**Introduction:**

Cross-country sit-skiers are often individuals with spinal cord injuries, cerebral palsy, or lower limb disabilities, relying heavily on upper limb strength to generate propulsion during skiing. However, frequent shoulder joint movements significantly increase the incidence of shoulder joint disorders. Therefore, quantifying muscle forces during movement is crucial for understanding upper limb force generation patterns. Currently, electromyography (EMG)-driven neuromusculoskeletal (NMSK) models are the predominant method for calculating muscle forces and joint moments. However, this approach heavily depends on the quality and quantity of EMG data. Surface electrodes are typically used to collect activity data from superficial muscles, but during dynamic movements, factors such as skin stretching, sweating, or friction may cause electrode detachment or poor contact, leading to EMG signal acquisition failures or data loss. In this study, we propose a synergy-assisted EMG-driven NMSK model to predict the activation patterns of missing muscles for cross-country sit-skiing double poling.

**Methods:**

This method is based on individualized EMG-driven NMSK models constructed for each participant, incorporating data from 10 muscles. By utilizing the activation data of 9 known muscles, the model predicts the activation of one missing muscle through synergy analysis. For synergy method selection, we systematically compared four approaches: Non-negative Matrix Factorization (NMF), Principal Component Analysis (PCA), Independent Component Analysis (ICA), and Factor Analysis (FA).

**Results:**

The results demonstrated NMF’s superior performance at 5 synergies, accurately predicting any missing muscle activation among 10 muscles ( r = 0.79 ± 0.25 vs. 0.14 ± 0.60-0.45 ± 0.63 for alternatives, p < 0.05), with lower errors (RMSE: 0.21 ± 0.11, p < 0.05 vs. ICA/FA, p < 0.1 vs. PCA; MAE: 0.17 ± 0.09, all p < 0.05).

**Conclusion:**

This finding validates the effectiveness of the proposed method in predicting upper limb muscle activation during coupled shoulder and elbow joint movements.

## 1 Introduction

Cross-country sit-skiing, as a high-intensity aerobic endurance sport, was officially included in the competition system at the 4th winter Paralympic Games held in Innsbruck, Austria, in 1988 (Gastaldi et al., 2012). Based on the skier’s ability to control their trunk and pelvis, the International Ski and Snowboard Federation (FIS) classifies athletes into five levels (LW10 to LW12), ranging from low to high. Athletes of all levels compete in the same race, with final rankings determined by adjusting their actual race times using level-specific time factors (FIS, 2024). Cross-country sit-skiers, commonly individuals with spinal cord injuries, growth defects, or cerebral palsy, utilize a sled-based double poling (DP) technique involving trunk flexion and coordinated upper limb muscle activation to generate propulsive force (Ohlsson and Laaksonen, 2017; Rosso et al., 2017; Chen et al., 2023). Existing research has shown that LW10 athletes can generate significant propulsive force during the early phase of the DP cycle by rapidly pressing down with their arms and swinging their upper body (Gastaldi et al., 2016). Liu et al., through an analysis of upper limb isokinetic muscle strength during the DP cycle, further confirmed the critical role of enhancing upper limb strength and coordination in improving athletic performance (Liu et al., 2022). However, studies on upper limb muscle strength in this sport remain relatively scarce. To date, only one study has examined the peak forces of six major muscle groups under different poling camber angles during the DP cycle (Tian et al., 2023).

Methods for quantifying muscle force fall into two primary categories: direct and indirect approaches. Direct measurement methods require surgical implantation of force sensors within human tissue to collect data. Although this approach provides precise measurements, its invasive nature carries inherent risks such as infection and tissue damage, limiting its application primarily to clinical research rather than widespread testing environments (Beidokhti et al., 2017; Trepczynski et al., 2018). Indirect measurement methods utilize neuromusculoskeletal (NMSK) modeling, which integrates computational approaches for calculating muscle activity with musculoskeletal geometries and contact models, to estimate muscle forces and joint moments during movement effectively (Rabbi et al., 2024). A fundamental challenge in this field stems from the anatomical complexity of the human musculoskeletal system: the number of muscles exceeds the skeletal degrees of freedom (DOFs), resulting in muscle redundancy. To resolve this redundancy, researchers have implemented optimization algorithms, notably static optimization (SO) and dynamic optimization (DO), for estimating muscle activation levels (Anderson and Pandy, 2001). These algorithms assume unique muscle force distribution patterns during movement and optimal muscle function. Nevertheless, without experimental electromyography (EMG) data (Manal and Buchanan, 2003) or established muscle contribution ratios (Ackermann and van den Bogert, 2010), these optimization methods cannot generate definitive solutions. Additionally, the predicted muscle activation patterns often fail to accurately represent physiological muscle activity or account for muscle co-contraction. Lloyd and Besier addressed these limitations by proposing an EMG-driven method that has gained widespread acceptance (Lloyd and Besier, 2003). This approach utilizes experimentally measured EMG data and musculotendon unit kinematic data from the NMSK model as inputs. The optimization objective minimizes the discrepancy between joint moments derived from inverse dynamics (ID) calculations and those obtained through EMG-driven model, thereby predicting muscle forces and joint moments (Buchanan et al., 2004). This approach successfully addresses muscle redundancy while facilitating muscle-tendon property calibration. However, the accuracy of muscle force calculations in the EMG-driven model critically depends on the reliability of the collected EMG data. While surface EMG devices are widely employed in biomechanics research due to their non-invasive nature and practical applicability, they cannot capture EMG data from deep muscles that significantly contribute to joint moments. Sartori et al. developed an optimization-assisted EMG-driven model to overcome limitations in EMG data collection from specific muscle groups (Sartori et al., 2014). In this approach, activation signals for muscles with experimentally measured EMG data are fine-tuned during the optimization process, while activation signals for muscles without EMG data are estimated entirely through SO. However, this method lacks robust validation of its predictions for unmeasured muscle activation against experimental data. Moreover, the SO approach, which processes individual time frames independently, may produce unrealistic discontinuities in muscle activation patterns.

To this end, Ao et al. proposed a muscle synergy extrapolation method to estimate missing muscle activation, enhancing the EMG-driven model’s muscle force calculations (Ao et al., 2020). The muscle synergy theory originates from Bernstein’s hypothesis of a dimensionality reduction control strategy, which was later validated and developed into a theory by Bizzi et al. through frog experiments and modeling (Bernshteĭn, 1967; Bizzi et al., 1991; Bizzi et al., 2008). This theory suggests that the central nervous system (CNS) simplifies complex motor control by coordinating the activation of functionally related muscle groups. Building on this theory, Ao et al. reduced the dimensionality of experimental EMG data into time-varying synergy activation coefficients and corresponding time-invariant synergy vectors. The synergy vectors define the contribution weights of each synergy activation coefficient to individual muscle activation. By incorporating the muscle synergy structure into the prediction of muscle activation, this method not only eliminates discontinuities between adjacent time frames but also reduces the number of design variables in the optimization process. In this paper, we refer to these approaches that leverage muscle synergy extrapolation for missing EMG estimation as “synergy-assisted EMG-driven NMSK modeling.” This synergy-assisted EMG-driven NMSK muscle force computation method has been successfully applied to muscle force predictions in gait and upper limb movements (Ao et al., 2022; Li et al., 2023; Tahmid et al., 2024). However, its potential application in scenarios involving complex shoulder and elbow joint movements remains insufficiently validated.

Therefore, this study aims to develop a synergy-assisted EMG-driven NMSK model tailored for the DP technique in cross-country sit-skiing, with the objective of predicting the activation of unmeasured upper limb muscles and selecting the optimal synergy extraction method for model assistance. Specifically, kinematic, kinetic, and surface EMG data were collected from participants during a 30-s maximal effort DP test. Based on these data, an EMG-driven NMSK model was constructed, with individualized parameter calibration and validation performed to ensure accurate muscle force estimation during the DP motion. To address the limitations of experimentally collected EMG data, this study employs muscle synergy analysis to reasonably predict the EMG signals of unmeasured muscles from a limited set of measured muscles.

## 2 Methods

**FIGURE 1:**
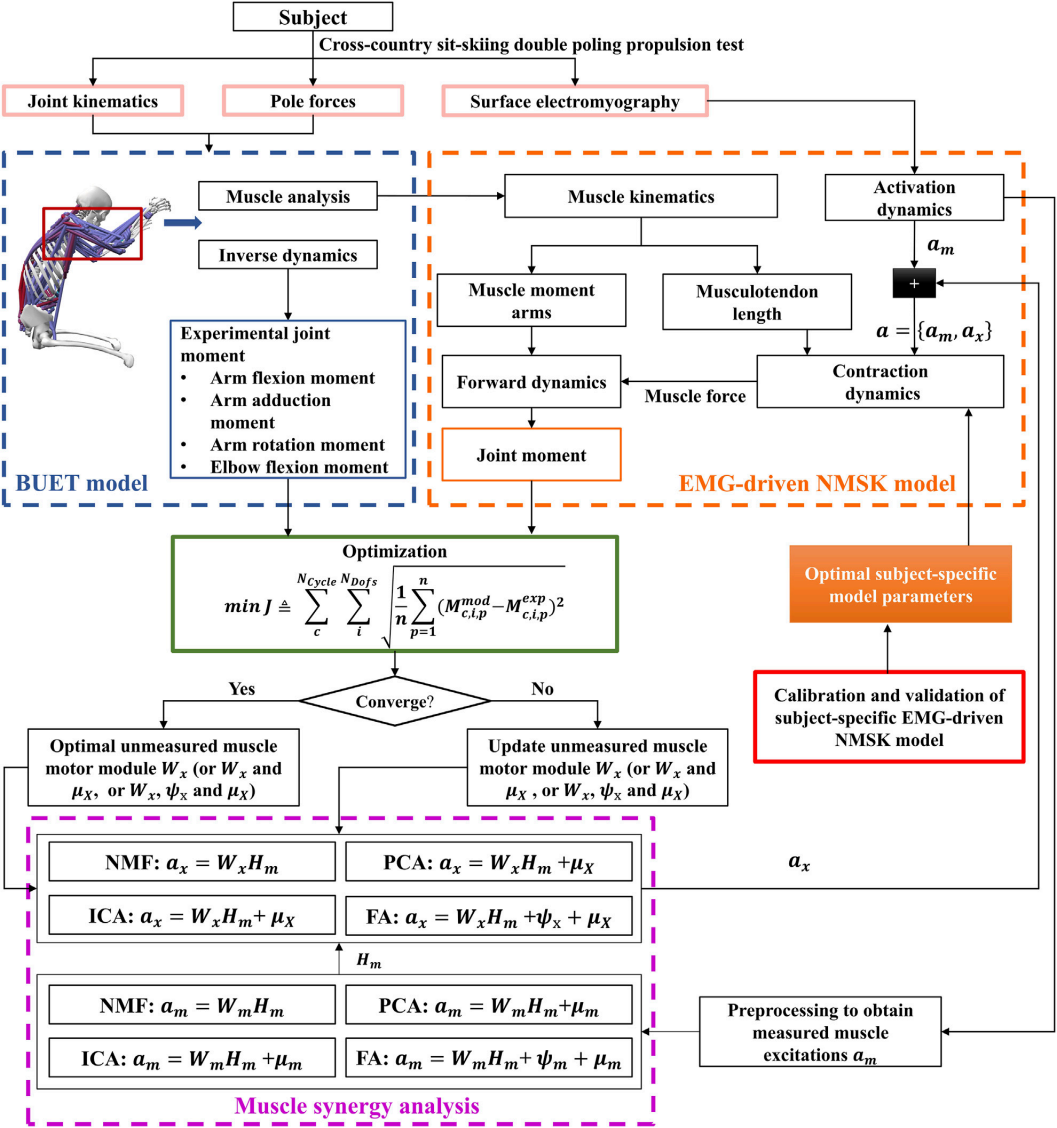
Flowchart of the synergy-assisted EMG-driven NMSK model method. The pink solid box section indicates data collection; the blue dashed box section denotes the NMSK model; the orange dashed box section is the EMG-driven NMSK model; the red solid box area represents the calibration and validation EMG-driven NMSK model; the purple dashed box part represents muscle synergy analysis; and the green solid box part illustrates the optimization process.

The following content will introduce a method for calculating muscle force using a synergy-assisted EMG-driven NMSK model from six aspects (Figure 1).

### 2.1 Participants

This study recruited three male college students from the Capital University of Physical Education and Sports as participants (age: 21 ± 2 years, weight: 76.67 ± 5.77 kg, height: 1.84 ± 0.09 m). All participants declare that they have no restrictions or pain in the shoulder, elbow, or trunk joints, indicating the absence of upper limb musculoskeletal disorders. This study was approved by the Ethics Committee of Capital University of Physical Education and Sports (Beijing, China) and conducted in accordance with relevant guidelines. All participants provided written informed consent, including consent for image publication where applicable.

### 2.2 Experimental data collection and processing

**FIGURE 2:**
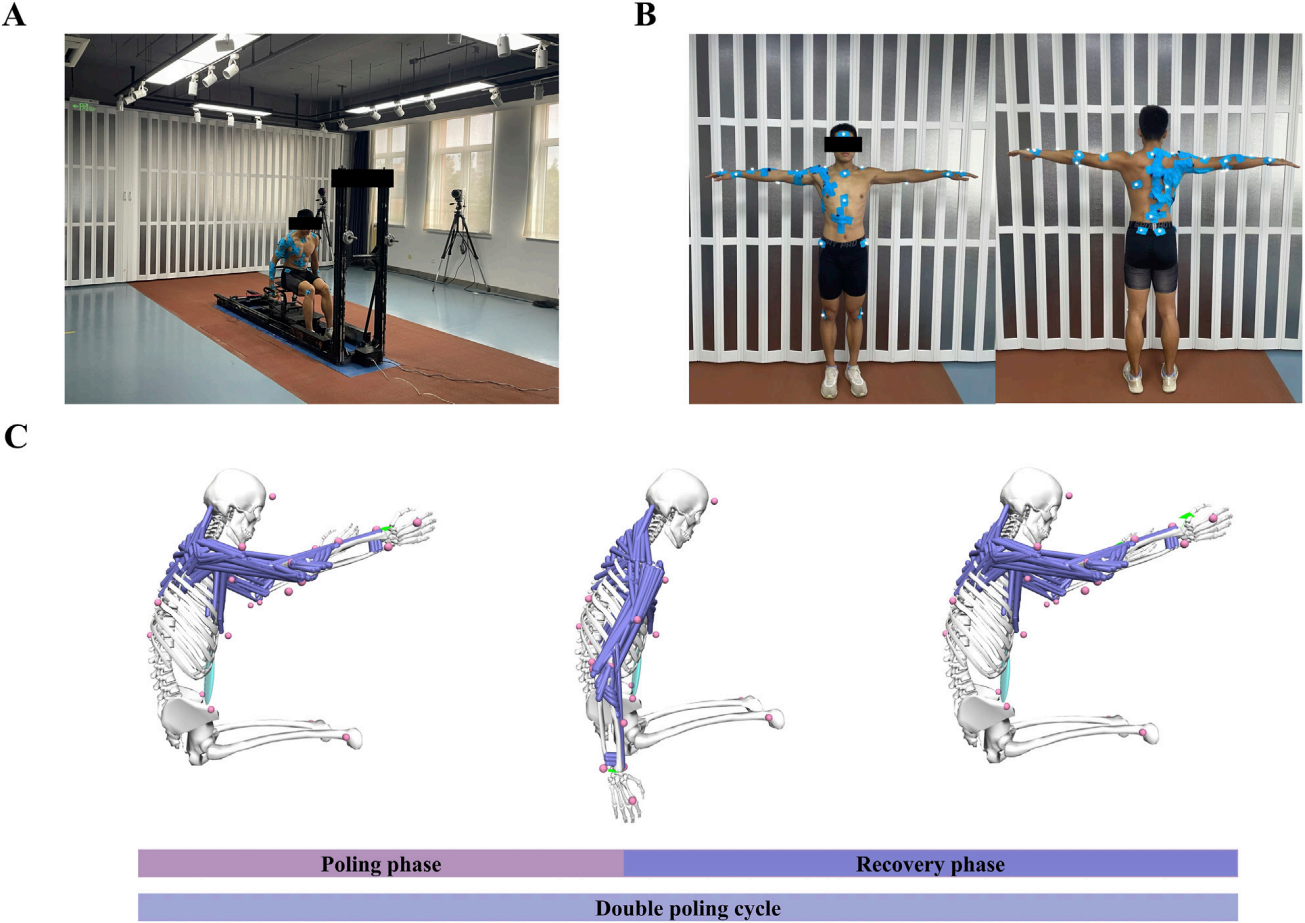
Experimental setup and simulation overview. **(A)** Subject performed DP on a cross-country sit-skiing smart training device. **(B)** Reflective marker placement and surface EMG setup were applied to the subject. **(C)** A simplified BUET model was used to simulate the DP, with the green arrows on the hands representing the application of pole forces. Abbreviations: double poling (DP), poling phase (PP), recovery phase (RP).

The participants were instructed to perform a 30-s maximum effort test on a self-developed cross-country sit-skiing smart training device (Liu et al., 2020; Liu et al., 2022) (Figure 2A). During this process, kinematic data, EMG signals, and bilateral pole forces were synchronized. Before testing, each participant was required to complete a 10-min warm-up at their preferred pace to familiarize themselves with the equipment. The rolling resistance was set to 5% of the participant’s body weight to simulate real double-poling skiing conditions. During the formal testing phase, participants were instructed to exert maximum effort throughout the entire test to ensure sufficient data collection. A minimum of 14 DP cycles was recorded for each test; tests yielding fewer than this number were discarded (Chen et al., 2023). A 10-min rest period was scheduled between each test to allow participants to recover and prevent muscle fatigue.

The three-dimensional trajectories were recorded using an optoelectronic camera system (Qualisys AB, Gothenburg, Sweden) sampling at 200 Hz. A total of 35 reflective markers were utilized, with 2 markers placed on each ski pole and 31 markers on the participants’ body (Figure 2B, for details refer to Chen et al. (Chen et al., 2023)). The kinematic data were filtered using a fourth-order Butterworth low-pass filter with a cutoff frequency of 10 Hz. The marker on the top of the pole was used to differentiate between the poling and recovery phases of the DP cycle. The poling phase (PP) began when the marker reached its highest position and ended at its lowest. Conversely, the recovery phase (RP) started at the marker’s lowest point and concluded when it returned to the highest position (Figure 2C).

Pole force was collected at 50 Hz using a uniaxial gauge load cell (Bengbu Zhongwan Sensor Co., Ltd, China) installed between the pole and the slider. The raw kinetic data were subsequently interpolated and smoothed using Matlab software. Two makers on each ski pole determined the direction of the pole force, with the point of force application defined as the midpoint between the two markers on the wrist, acting on the NMSK model’s hand (Figure 2C).

Surface EMG signals (Cometa Systems Co., Ltd, Italy) were collected with a sampling frequency of 2000 Hz (Figure 2B). Ten muscles (as shown in Table 1) on the right side of the body (subject’s dominant arm) were measured. EMG signals were integrated by a bandpass filter ranging from 50 to 300 Hz, then processed with full-wave rectification, linearly enveloped, and finally normalized. The normalization process involved scaling the EMG amplitudes of each muscle by the maximum EMG activation recorded for that specific muscle across all experimental trials (Bianco et al., 2018; Ao et al., 2020). Then each DP cycle was time-normalized to 100 points, with 50 points corresponding to the PP and the remaining 50 representing the RP.

**TABLE 1:** Moment arm functions of muscle in shoulder and elbow joint movement.

Experimental muscle EMG (abbreviation)	Arm flexion-extension moment arm	Arm adduction-abduction moment arm	Arm internal-external rotation moment arm	Elbow flexion-extension moment arm
Biceps brachii (BB)	Positive	N/A	N/A	Positive
Triceps brachii (TB)	Negative	Positive	N/A	Negative
Anterior deltoid (AD)	Positive	Negative	Positive	N/A
Middle deltoid (MD)	N/A	Negative	N/A	N/A
Posterior deltoid (PD)	Negative	Negative	Negative	N/A
Infraspinatus (IF)	Negative	Positive	Negative	N/A
Teres major (TMj)	Negative	Positive	Positive	N/A
Latissimus dorsi (LD)	Negative	Positive	Positive	N/A
Pectoralis major (PM)	Positive	Positive	Positive	N/A
Brachioradialis (BRD)	N/A	N/A	N/A	Positive

Arm flexion, adduction, internal rotation and elbow flexion are positive.

### 2.3 Development of EMG-driven NMSK model

The EMG-driven NMSK model comprises four components: the NMSK model for calculating muscle kinematics and reference moments, activation dynamics, contraction dynamics, and forward dynamics, as detailed below.

The NMSK model was based on the OpenSim BUET model developed for cross-country sit-skiing DP propulsion (Chen et al., 2023). The BUET model includes 17 articulating rigid bodies, 35 DOFs, and 472 musculotendon actuators. The glenohumeral and sternoclavicular joints were modeled as three-degrees-of-freedom rotational joint, which simulated the movement of the joints in the coronal, sagittal, and transverse planes. The acromioclavicular joint was established as a weld joint, but it was unable to move. Based on the BUET model, individualized models were scaled for each subject, with inverse kinematics calculations first performed to obtain joint angle during DP propulsion (Supplementary Figure S2), followed by ID and muscle analysis to obtain reference moments at the shoulder and elbow, as well as kinematic data of the muscles (musculotendon length and moment arm) during DP propulsion. Finally, the obtained data underwent time normalization, dividing one DP cycle into 100 points, with 50 designated for PP and the other 50 for RP. To reduce optimization time, we have reduced the number of musculotendon actuators in the BUET model to 124 (Figure 2C).

The activation dynamics model consists of two components: the backward difference model (as shown in Equation 1) and the nonlinear model (as shown in Equation 2) (Lloyd and Besier, 2003). The preprocessed EMG signals are converted to neural excitation using a critically damped, second-order dynamic system, and muscle activation is then computed via a nonlinear transformation. The respective formulations are as follows:

$$u\left(t\right)=\alpha e\left(t-d\right)-\left(C_{1}+C_{2}\right)u\left(t-1\right)-C_{1}C_{2}u\left(t-2\right)$$ (1)

$$a\left(t\right)=\frac{e^{Au\left(t\right)}-1}{e^{A}-1}$$ (2)

where all variables and parameters are as previously defined (see (Lloyd and Besier, 2003), for details).

The calculation of muscle force in muscle contraction dynamics is based on the following key parameters: maximum isometric force ( $F_{0}^{m}$ ), optimal fiber length ( $l_{0}^{m}$ ), tendon slack length ( $l_{s}^{t}$ ), musculotendon length ( $L^{MT}$ ), along with the muscle activation ( a ) generated by the activation dynamics model. Within this computational framework, the Hill-type muscle model is employed to describe the mechanical properties of muscles (Equation 3) (Zajac, 1989) (Supplementary Figure S1A). The specific mechanical relationship formulas are as follows:

$$F^{MT}=F^{T}=F^{M}\cos\alpha =\left(F^{CE}+F^{PE}\right)\cos\alpha$$ (3)

$F^{MT}$ represents musculotendon force; $F^{T}$ represents tendon force; $F^{M}$ represents muscle force.

Active muscle force-activation-length-velocity relationship as shown in in Table 2 (Thelen, 2003). A Gaussian function was employed to model the active force-length relationship ( $f_{l}$ ), as shown in Supplementary Figure S1B. The parameter b varies according to the muscle fiber contraction state whether it undergoes shortening or lengthening phases (Supplementary Figure S1C). Passive muscle force-length relationship is represented by an exponential function as shown in Supplementary Figure S1B (Thelen, 2003). Tendon length-force relationship is represented by an exponential function as shown in Supplementary Figure S1D (Thelen, 2003; Ma et al., 2016). All mathematical formulations and specific parameter details are provided in Table 2.

**TABLE 2:** Parameters for musculotendon force and joint moment calculations.

Model components		Parameter	Definition	Value
Contraction dynamics	Active force-length relationship $\begin{array}{l} {\bar{F}}^{CE}=\frac{V^{m}\cdot b}{V_{\max}^{m}\cdot \left(0.25+0.75a\right)}+af_{l}\\ b=\left\{\begin{array}{l} af_{l}+\frac{{\bar{F}}^{CE}}{A_{f}};\\ {\bar{F}}^{CE}\leq af_{l}\\ \frac{\left(2+\frac{2}{A_{f}}\right)\left(af_{l}{\bar{F}}_{len}^{M}-{\bar{F}}^{CE}\right)}{\left({\bar{F}}_{len}^{M}-1\right)};\\ {\bar{F}}^{CE}>af_{l} \end{array}\right. \end{array}$ $\begin{array}{l} f_{l}=e^{\frac{-{\left({\bar{L}}^{M}-1\right)}^{2}}{\gamma }} \end{array}$	${\bar{F}}^{CE}$ = $F^{CE}/F_{0}^{m}$	Normalised active muscle force = active muscle force/maximum isometric force	
		$\bar{V^{m}}$ = $V^{m}/V_{\max}^{m}$	Normalised muscle fiber velocity = muscle fiber velocity/maximum contraction velocity	$V_{\max}^{m}$ = 10
		$L^{M}$ = $\left(L^{MT}-L^{T}\right)/\cos\alpha$	Muscle length = (musculotendon length - tendon length)/ cos⁡α ( α is pennation angle)	
		${\bar{L}}^{M}$ = $L^{M}/l_{0}^{m}$	Normalized muscle fiber length = muscle fiber length/optimal fiber length	
		${\bar{F}}_{len}^{M}$	Maximum normalized muscle force achievable when the fiber is lengthening	1.8
		$A_{f}$	Force-velocity shape factor	0.3
		γ	Shape factor	0.6
	Passive force–length relationship ${\bar{F}}^{PE}=\frac{e^{\frac{K^{PE}\left({\bar{L}}^{M}-1\right)}{\varepsilon_{0}^{M}}-1}}{e^{K^{PE}}-1}$	${\bar{F}}^{PE}=F^{PE}/F_{0}^{m}$	Normalised passive muscle force = passive muscle force/maximum isometric force	
		$K^{PE}$	Exponential shape factor	5
		$\varepsilon_{0}^{M}$	Passive muscle strain due to maximum isometric force	0.6
	Tendon length–force relationship $\begin{array}{l} \bar{l_{t}}=\frac{l_{s}^{t}\cdot \varepsilon_{toe}^{T}\cdot }{k_{toe}\cdot \log\left(\bar{F^{T}}\cdot \left(\frac{e^{k_{toe}}-1}{F_{toe}^{T}}\right)+1\right)}+l_{s}^{t};\\ \bar{F^{T}}\leq 0.33\\ \bar{l_{t}}=\left(\frac{\left(\bar{F^{T}}-\bar{F_{toe}^{T}}\right)}{k_{lin}}+\varepsilon_{toe}^{T}\right)\cdot l_{s}^{t}+l_{s}^{t};\\ \bar{F^{T}}>0.33 \end{array}$	$\bar{F^{T}}$ = $F^{T}/F_{0}^{m}$	Normalised tendon force = tendon force/maximum isometric force	
		$\bar{l_{t}}=L^{T}/l_{s}^{t}$	Normalised tendon length = tendon length/tendon slack length	
		$F_{toe}^{T}$	Tendon force at the transition from nonlinear to linear behavior	
		$\bar{F_{toe}^{T}}$ = $F_{toe}^{T}/F_{0}^{m}$	Normalised $F_{toe}^{T}$ = $F_{toe}^{T}$ /maximum isometric force	0.33
		$\varepsilon^{T}$	Tendon strain	
		$\varepsilon_{0}^{T}$	Tendon strain due to maximum isometric force	0.04
		$\varepsilon_{toe}^{T}$	Tendon strain threshold for linear behavior	0.609 $\varepsilon_{0}^{T}$
		$k_{toe}$	Exponential shape factor	3
		$k_{lin}$	Linear scale factor	1.712/ $\varepsilon_{0}^{T}$
Forward dynamics $M_{i}=\sum_{j}^{N_{muscle}}F_{0,j}^{m}\cdot \left[{\bar{F}}_{j}^{CE}+{\bar{F}}_{j}^{PE}\right]\cdot r_{i,j}^{m}\cdot \cos\alpha_{j}$		$M_{i}$	Joint moment of the i th joint	
		$r_{i,j}^{m}$	Moment arm of the j th muscle relative to the i th joint	
Objective function of the calibration model		$M_{c,i,p}^{\mathrm{mod}}$	Model-predicted moment at the p th data point of the i th joint in the c th cycle	
		$M_{c,i,p}^{\exp}$	ID-calculated moment at the p th data point of the i th joint in the c th cycle	
		n	Total number of data points in each cycle	100
		$N_{cyc}$	Total number of cycles	5
		$N_{dof}$	Total number of degrees of freedom	4

After completing the force calculations for contraction dynamics, the joint moments are derived using the principles of forward dynamics. The resultant moment at a single joint is equal to the sum of the products of all muscle forces acting on that joint, their corresponding pennation angle cosines, and the moment arms (details are in Table 2).

**FIGURE 3:**
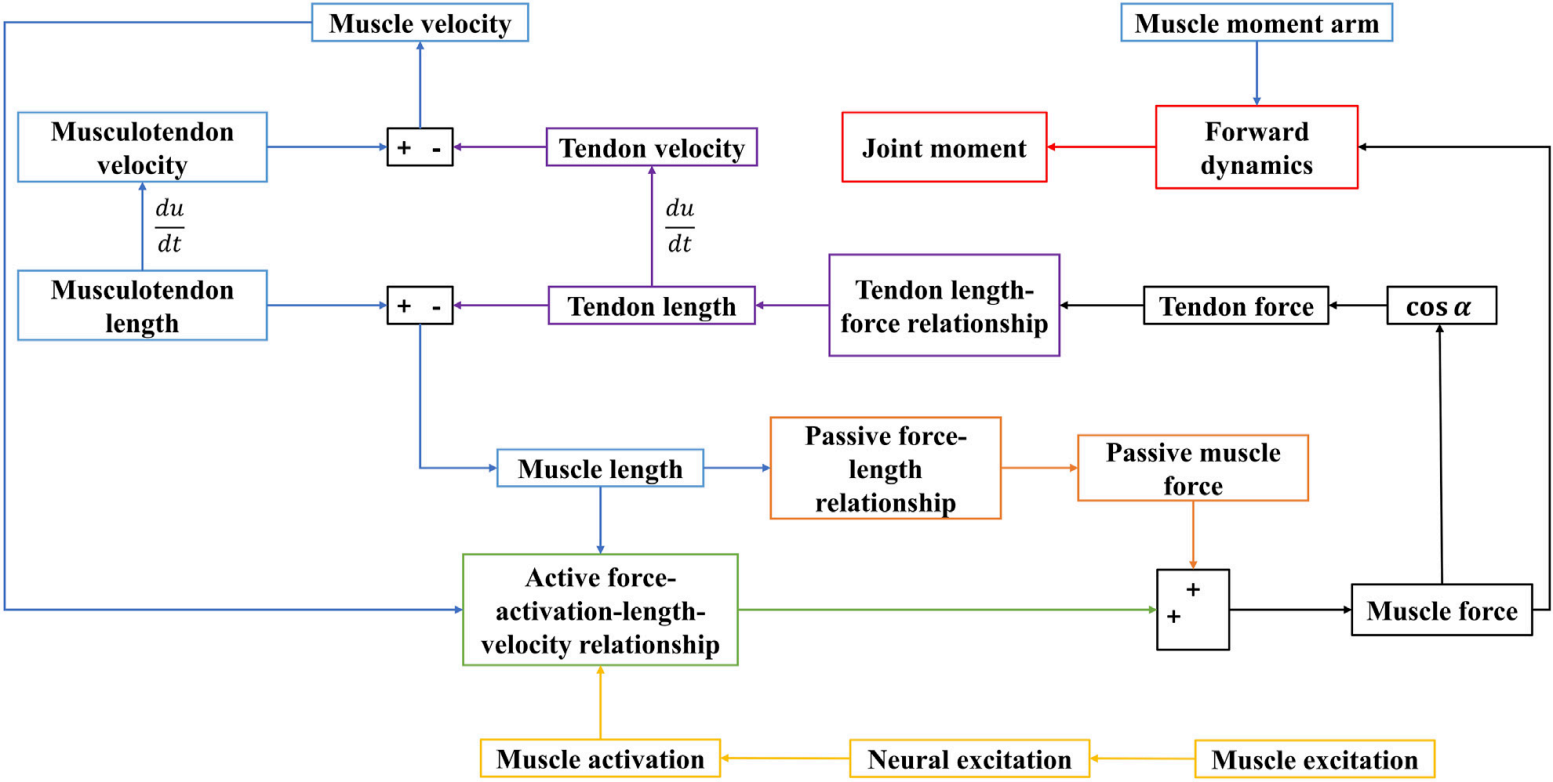
Dynamic calculation process of the EMG-driven model. Blue represents the muscle kinematics model; yellow represents the activation dynamics model; black represents the mechanical relationship model between muscle and tendon; green represents the active muscle force-activation-length-velocity relationship mode; orange represents the passive muscle force-length relationship model; purple represents the tendon length-tendon force relationship model; red represents the forward dynamics model.

Utilizing these four core components, the EMG-driven NMSK model was developed. The dynamic computation process of this model is illustrated in Figure 3, and the algorithm was implemented within the Matlab environment.

### 2.4 Calibration and validation EMG-driven NMSK model

After constructing the EMG-driven NMSK model, it is necessary to calibrate individualized parameters for each subject. Referring to previous research, Kian et al. successfully calibrated the EMG-driven model for shoulder motion tasks by optimizing musculotendon parameters $F_{0}^{m}$ , $l_{0}^{m}$ , $l_{s}^{t}$ , and activation dynamics coefficients A , $C_{1}$ , $C_{2}$ (Kian et al., 2021). Furthermore, the findings of Blache et al. demonstrated that muscle force estimation for the shoulder and upper limb is highly sensitive to variations in musculotendon properties, with the variability of $F_{0}^{m}$ and $l_{0}^{m}$ having the most significant impact (Blache et al., 2019). Based on these two studies, we selected $F_{0}^{m}$ , $l_{0}^{m}$ , $l_{s}^{t}$ , A , $C_{1}$ , $C_{2}$ , and d as the parameters to be calibrated to achieve individualized settings for each subject.

The calibration process is as follows. Firstly, kinematic data, external force data, and EMG signals from 5 DP cycles were input into the EMG-driven model to calculate the joint moments of the shoulder and elbow. Subsequently, the model-predicted moments were compared with those obtained through ID. A simulated annealing algorithm was employed for iterative optimization, continuously adjusting the model parameters to minimize the root mean square error (RMSE) between the two sets of results (as shown in Equation 4; Table 2). Finally, individualized parameters for each subject were determined and output, ensuring that the model accurately captures inter-individual physiological differences. It is worth noting that for the parameters $F_{0}^{m}$ , $l_{0}^{m}$ , and $l_{s}^{t}$ , we allowed them to vary within ± 50% of their initial values, which were derived from the standard settings of a generic model. Additionally, the initial values of parameters A , $C_{1}$ , $C_{2}$ , and d were set to −0.1, 0.5, −0.5, and 0.05, respectively, and were adjusted within the ranges of −3 to 0, −1 to 1, −1 to 1, and 0.01 to 0.3. This parameter configuration not only ensures the model’s high sensitivity and accuracy in capturing individual differences but also guarantees the physiological plausibility and interpretability of the parameters.

$$\min J≜\sum_{c}^{N_{Cycle}}\sum_{i}^{N_{Dofs}}\sqrt{\frac{1}{n}\sum_{p=1}^{n}{\left(M_{c,i,p}^{\mathrm{mod}}-M_{c,i,p}^{\exp}\right)}^{2}}$$ (4)

Using 5 DP cycles data that differ from the calibration process, the individualized EMG-driven NMSK model of the subjects was validated by comparing the moment results calculated by the ID.

### 2.5 Synergy-assisted EMG-driven NMSK model

Based on the calibrated and validated subject-specific EMG-driven NMSK model, a synergy-assisted EMG-driven NMSK model was developed (Figure 1). In this study, the EMG data of one muscle were sequentially excluded and treated as unmeasured, while the remaining muscles’ EMG data were treated as measured. The measured EMG data were organized into a matrix $e_{m}$ , which was input into the activation dynamics model to obtain the muscle activation matrix $a_{m}$ . This matrix $a_{m}$ was used as input for both the EMG-driven model and muscle synergy analysis.

Non-negative Matrix Factorization (NMF) was applied to decompose $a_{m}$ into two matrices: the motor module $W_{m}$ and the motor primitive $H_{m}$ , according to $V\approx V_{R}=WH$ . The matrix $W_{m}$ represents the relative weights of individual muscles ( m×l ), where l denotes the number of synergies, and $H_{m}$ represents the time-dependent coefficients ( l×n ). To investigate the effect of the number of synergies on predicting unmeasured muscle activations, l was predefined in the NMF algorithm. Since extracting a number of synergies equal to the total number of measured EMG signals does not reduce the dimensionality of the data, the maximum number of synergies was restricted to 75% of the total number of muscles. In this study, synergy numbers range from 2 to 7 were evaluated. For each synergy number, the factorization process was repeated 10 times with newly randomized initial matrices $W_{m}$ and $H_{m}$ in each iteration to avoid local minima. During this process, the coefficient of determination ( $R^{2}$ ) between the reconstructed ( $V_{R}$ ) and original activation matrices ( V ) was calculated, and the solution with the highest $R^{2}$ was selected for each synergy number. Subsequently, based on the synergy weight matrix $W_{x}$ containing the relative weights of unmeasured muscles ( 1×l ), combined with a matrix $H_{m}$ extracted from the measured muscle synergy analysis, the activation of unmeasured muscles $a_{x}$ was reconstructed ( $a_{x}=W_{x}H_{m}$ ). Notably, the matrix $W_{x}$ was obtained through optimization iterations by tracking the experimental joint moments. During this process, the values of $W_{x}$ were constrained to be greater than 0, while the reconstructed muscle activation values ( $a_{x}$ ) were restricted to a range between 0 and 1. Furthermore, this study systematically investigated the efficacy of Principal Component Analysis (PCA), Independent Component Analysis (ICA), and Factor Analysis (FA) in extracting synergistic patterns for predicting muscle activation accuracy. Through comparative analysis of the predictive performance among these four methods (including NMF), we aimed to identify the optimal synergistic assistance approach for accurate upper-limb muscle activation prediction in shoulder-elbow coupled movements, thereby enhancing the predictive robustness of the synergy-assisted EMG-driven NMSK model (detailed methodology provided in Supplementary Material).

In this section, the synergy-assisted EMG-driven NMSK model was evaluated using data from 10 DP cycles, which were the same datasets used for calibrating and validating the EMG-driven NMSK model. The entire process was implemented in MATLAB, utilizing a simulated annealing algorithm to optimize the objective function. The predicted muscle activation values were then compared with the experimentally measured results.

### 2.6 Statistical analyses

To evaluate the performance of the synergy-assisted EMG-driven NMSK model in predicting unmeasured muscle activation, this study selected 10 complete DP cycles from each participant during the calibration and validation phases of the EMG-driven NMSK model for comprehensive evaluation. First, $R^{2}$ was calculated to quantify and evaluate the ability of NMF to reconstruct muscle activation patterns under different synergy number conditions. Furthermore, to further assess the overall performance of the synergy-assisted EMG-driven NMSK model in predicting muscle activations and joint moments, as well as the specific impact of different synergy numbers on the prediction results, the following metrics were calculated: (1) %RMSE (as in Equation 5), r , and %MAE (as in Equation 6) between the model-predicted joint moments and the reference joint moments obtained from ID, with paired t-tests used for statistical comparison; and (2) RMSE, r and MAE between the predicted unmeasured muscle activation and the experimentally measured muscle activation (all synergy-assisted methods). Statistical analyses were conducted using the Kruskal–Wallis test, followed by Dunn’s test for post hoc comparisons. All analyses were performed in Matlab, with the significance levels set at p < 0.05.

$$\%RMSE=\frac{\sqrt{\frac{1}{n}{\sum_{p=1}^{n}\left(M_{p}^{\exp}-M_{p}^{\mathrm{mod}}\right)}^{2}}}{\max\left(M^{\exp}\right)-\min\left(M^{\exp}\right)}$$ (5)

$$\%MAE=\frac{\frac{1}{n}\sum_{p=1}^{n}\left|M_{p}^{\exp}-M_{p}^{\mathrm{mod}}\right|}{\max\left(M^{\exp}\right)-\min\left(M^{\exp}\right)}$$ (6)

## 3 Results

### 3.1 Assessment of EMG-driven NMSK model accuracy

**FIGURE 4:**
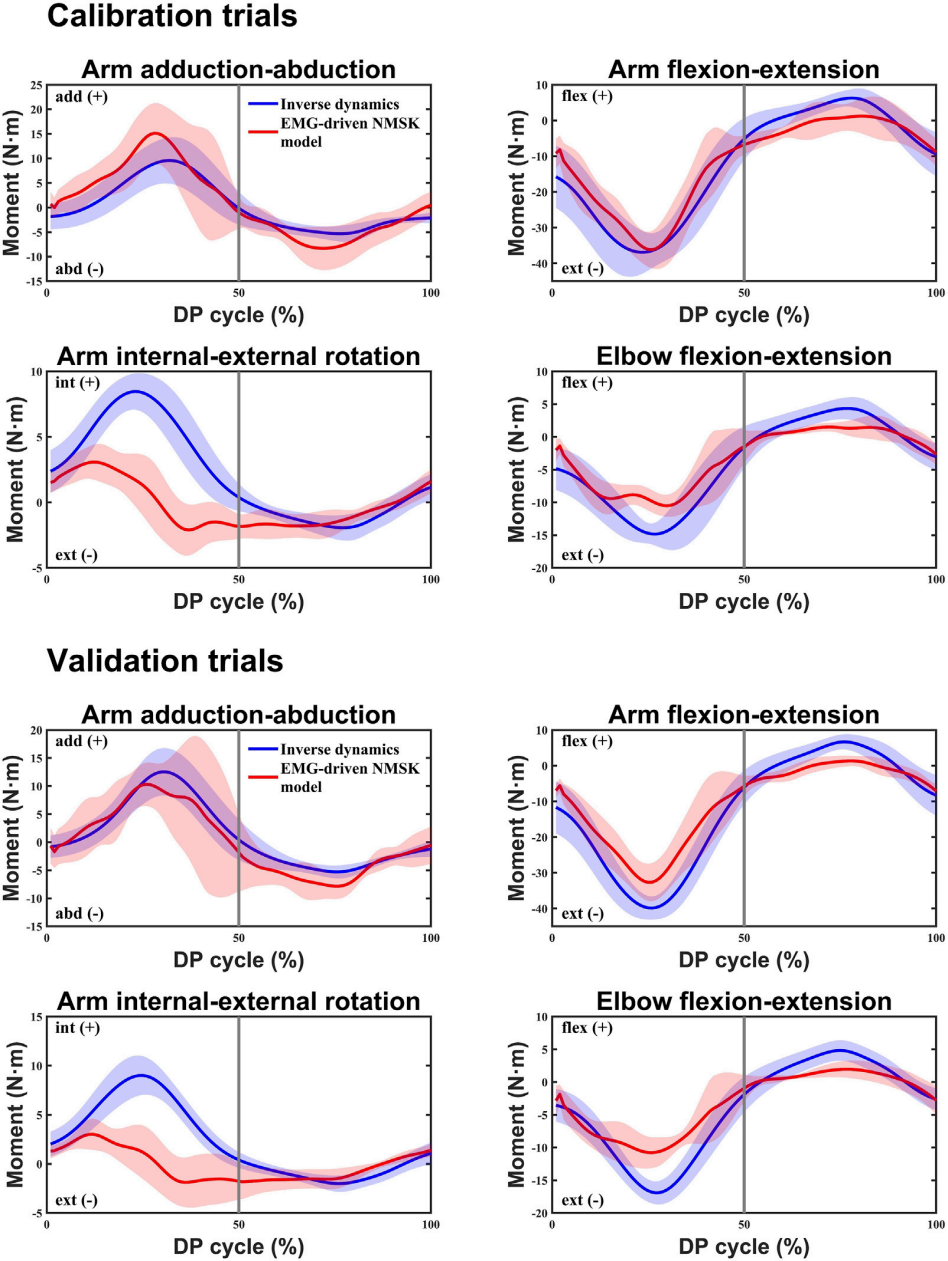
In both the calibration and validation trials, the inverse dynamics (ID) method and the EMG-driven NMSK model with all muscles were used to calculate the average joint moments for four degrees of freedom at the shoulder and elbow joints of three participants. The blue curve represents the average reference joint moments calculated by the ID method, while the red curve represents the average joint moments calculated by the EMG-driven NMSK model. The 0%–50% of the cycle corresponds to the poling phase (PP) of the double poling (DP), while the 50%–100% corresponds to the recovery phase (RP).

Figure 4 illustrated the relationship between the moment calculations of the EMG-driven NMSK model and the experimental joint moments calculated using ID for three subjects during calibration and validation trials. The figure shows that, aside from a slight shortfall in the accuracy of predicting the arm internal-external rotation moment, the predictions for other joint moments demonstrate a high level of accuracy. Further analysis of the data in Table 3 revealed that during the calibration trials, the model performed best in the arm flexion-extension moment (%RMSE = 0.15 ± 0.03, r > 0.92) and elbow flexion-extension moment (%RMSE = 0.17 ± 0.02, r > 0.92). In contrast, the prediction accuracy for the arm adduction-abduction moment and internal-external rotation moment was relatively lower, particularly for internal-external rotation moment, which had a r of only 0.55 ± 0.14. In the validation trials, an independent set of five DP cycles was used as the validation dataset, and paired t-test results indicated no significant statistical difference between the calibration and validation data. During the validation trials, the arm adduction-abduction moment, flexion-extension moment, and elbow flexion-extension moment all maintained high correlation ( r > 0.9). However, it was noteworthy that the r for the arm internal-external rotation decreased from 0.55 to 0.43, reflecting the uncertainty in the prediction for this degree of freedom (DOF). The relatively poor estimation of internal and external rotation moments is likely attributable to the absence of deep rotator cuff muscles in the EMG-driven NMSK model. Due to the limitations of our study, only EMG data from ten superficial muscles were collected.

**TABLE 3:** The %RMSE, r and %MAE between experimental joint moments and EMG-driven predicted in calibration trials and validation trials.

Degree of freedom	Calibration trials			Validation trials		
	%RMSE	r	%MAE	%RMSE	r	%MAE
Arm adduction-abduction	0.36 ± 0.18	0.78 ± 0.20	0.27 ± 0.14	0.31 ± 0.06	0.74 ± 0.15	0.23 ± 0.05
Arm flexion-extension	0.15 ± 0.03	0.92 ± 0.05	0.12 ± 0.03	0.17 ± 0.03	0.91 ± 0.06	0.14 ± 0.02
Arm internal-external rotation	0.36 ± 0.08	0.55 ± 0.14	0.27 ± 0.07	0.38 ± 0.13	0.43 ± 0.28	0.28 ± 0.10
Elbow flexion-extension	0.17 ± 0.02	0.92 ± 0.04	0.13 ± 0.02	0.17 ± 0.04	0.90 ± 0.08	0.14 ± 0.03

### 3.2 Muscle activation prediction

**FIGURE 5:**
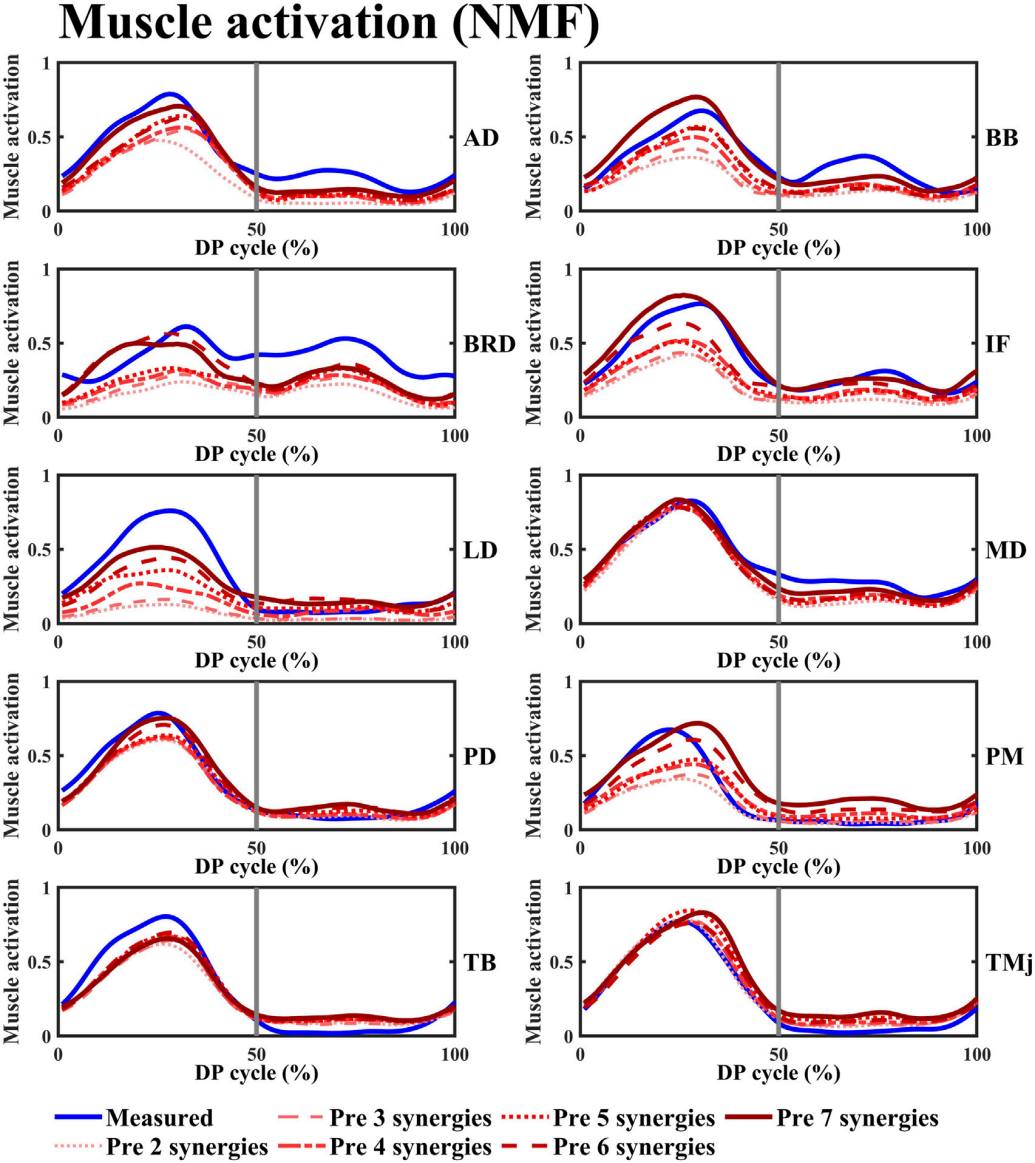
The variation curves of the predicted average space missing muscle activation and the experimentally measured average muscle activation under different synergy number conditions were plotted. The blue curve represents the experimentally measured values, while the red curve corresponds to the predicted values calculated using the NMF synergy-assisted EMG-driven NMSK model. The 0%–50% of the cycle corresponds to the poling phase (PP) of the double poling (DP), while the 50%–100% corresponds to the recovery phase (RP). Muscle abbreviations: AD, anterior deltoid; BB, biceps brachii; BRD, brachioradialis; IF, infraspinatus; LD, latissimus dorsi; MD, middle deltoid; PD, posterior deltoid; PM, pectoralis major; TB, triceps brachii; TMj, teres major.

**FIGURE 6:**
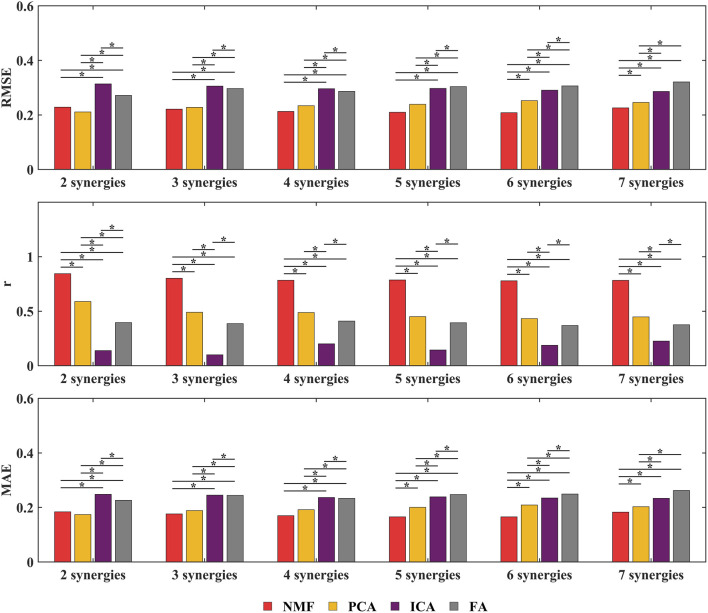
Overall RMSE, r , and MAE between the predicted missing muscle activations by the synergy-assisted EMG-driven NMSK model using different synergistic assistance methods under varying synergy numbers and the experimental measurements. Red represents NMF, yellow represents PCA, purple represents ICA, gray represents FA. * indicates significant differences between groups ( p < 0.05). Muscle abbreviations: AD, anterior deltoid; BB, biceps brachii; BRD, brachioradialis; IF, infraspinatus; LD, latissimus dorsi; MD, middle deltoid; PD, posterior deltoid; PM, pectoralis major; TB, triceps brachii; TMj, teres major.

This study employed NMF, PCA, ICA, and FA synergy-assisted EMG-driven NMSK models for muscle activation prediction, with results shown in Figures 5, 6 and Supplementary Figures S3–S9. The findings revealed that the number of synergies had no significant impact on RMSE, r , and MAE for missing muscles in PCA, ICA and FA methods, except for TB and TMj muscles, where statistically significant differences in these metrics were observed with 2 synergies. Critically, none of these three methods could reliably provide satisfactory predictions for all possible single-muscle missing scenarios. Specifically: the PCA approach lacked a single synergy number that could simultaneously maintain moderate correlations ( r > 0.35) for muscles BB, BRD, IF, LD, and PM, with negative correlations even observed (Supplementary Figures S3–S4; Supplementary Table S1); the ICA method demonstrated limited predictive capability across all single-muscle scenarios, with r < 0.51 and RMSE values averaging approximately 0.3, indicating ineffective prediction performance (Supplementary Figures S5–S6; Supplementary Table S2); and the FA method poor predictive performance for muscles BB, BRD, IF, LD, and PM ( r < 0.35, RMSE >0.3; Supplementary Figures S7–S8; Supplementary Table S3). Statistical analysis of RMSE, r and MAE between predicted and experimental data across six synergy numbers confirmed that the NMF method consistently outperformed other methods under all synergy conditions, exhibiting the smallest prediction errors (RMSE: 0.21 ± 0.11 to 0.23 ± 0.13; MAE: 0.17 ± 0.09 to 0.18 ± 0.13; p < 0.05 vs. ICA/FA; both RMSE and MAE showing partial non-significance vs. PCA) and highest correlations ( r : 0.78 ± 0.26 to 0.84 ± 0.23, p < 0.05 vs. PCA/ICA/FA) (Figure 6; Supplementary Figure S9). Given these findings, we focus our subsequent detailed analysis exclusively on the NMF method results, which demonstrate superior performance in predicting muscle activation patterns under various single missing muscle conditions.

**FIGURE 7:**
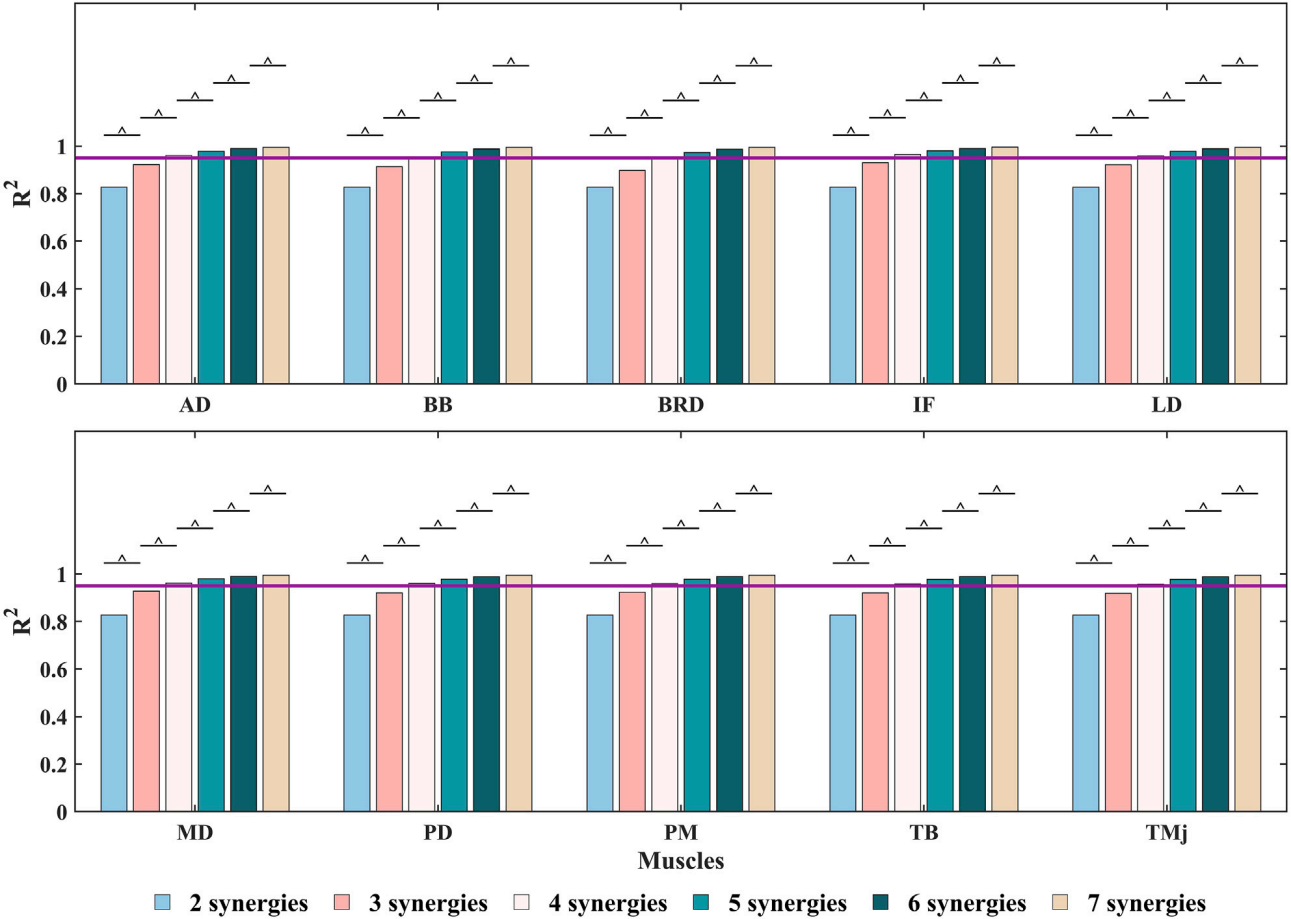
The $R^{2}$ value was calculated to evaluate the ability of the NMF method to reconstruct the measured muscle activation matrix after the removal of a specific muscle’s EMG data under different synergy numbers. The purple line represents $R^{2}$ = 0.95. ^ indicates no significant differences between the groups ( p > 0.05). Muscle abbreviations: AD, anterior deltoid; BB, biceps brachii; BRD, brachioradialis; IF, infraspinatus; LD, latissimus dorsi; MD, middle deltoid; PD, posterior deltoid; PM, pectoralis major; TB, triceps brachii; TMj, teres major.

The NMF synergy-assisted EMG-driven NMSK model demonstrates excellent performance in predicting missing muscle activation (as shown in Figure 5). The predicted muscle activation curves closely resemble the experimental measurement curves, effectively capturing the dynamic changes in muscle activation, particularly reflecting the actual trends during both the rising and falling phases of activation. In the early phase of the DP cycle (within the PP), the model’s predicted curves show a high degree of overlap with the experimental values, indicating its accuracy during the initial activation stage. The variation in the number of synergies affects the prediction of muscle activation. When the number of synergies reaches four or more, the $R^{2}$ value exceeds 0.95, indicating that the reconstruction effect is very satisfactory (Figure 7).

**FIGURE 8:**
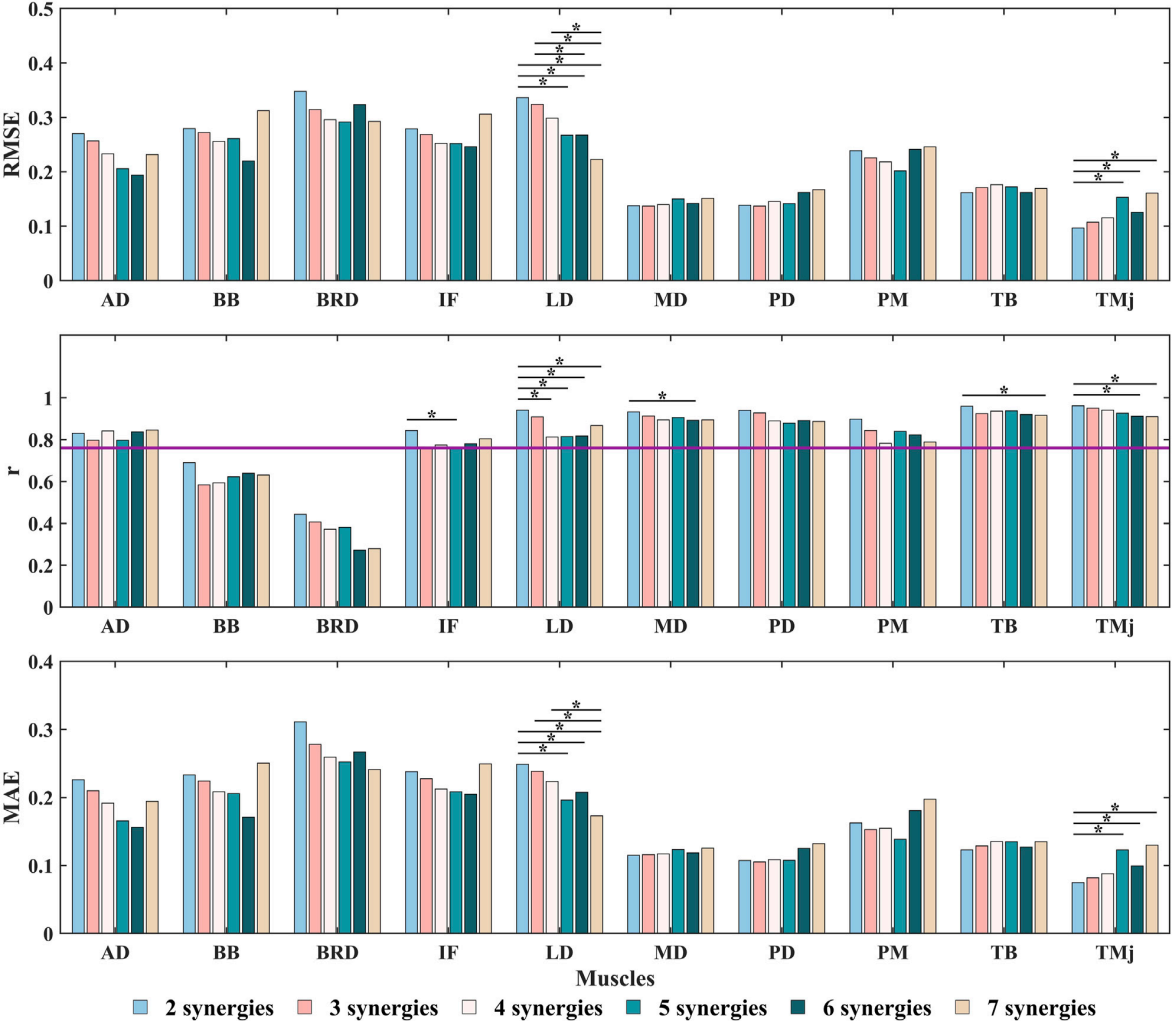
The RMSE, r , and MAE between the predicted missing muscle activations by the NMF synergy-assisted EMG-driven NMSK model under different synergy numbers and the experimental measurements. The purple line represents r = 0.76. * indicates significant differences between groups ( p < 0.05). Muscle abbreviations: AD, anterior deltoid; BB, biceps brachii; BRD, brachioradialis; IF, infraspinatus; LD, latissimus dorsi; MD, middle deltoid; PD, posterior deltoid; PM, pectoralis major; TB, triceps brachii; TMj, teres major.

Further analysis using the Kruskal–Wallis test revealed the impact of the number of synergies on the RMSE, r , and MAE for predicting missing muscle activation (Figure 8; Supplementary Table S4). The results show that as the number of synergies increases, the RMSE and MAE for most muscles tend to decrease gradually, particularly between synergy counts of 2 and 5. However, when the number of synergies reaches 6 and 7, the RMSE for some muscles (such as BRD, PD, and PM) slightly increases, indicating a degree of instability. This suggests that when the number of synergies exceeds 5, the improvement in error metrics becomes very limited. Additionally, the RMSE and MAE for the LD and TMj muscles significantly decrease and increase, respectively, as the number of synergies increases, indicating that different muscles exhibit varying sensitivities to the number of synergies. The r values generally remain high (above 0.76 for all muscles except BB and BRD, indicating strong correlations), but for muscles IF, LD, MD, TB, and TMj, the r values significantly decrease as the number of synergies increases, with optimal values occurring at a synergy count of 2. This suggests that in certain cases, increasing the number of synergies may lead to a decline in the model’s fit. In summary, a synergy count of 5 is considered the optimal choice, as it allows the model to effectively capture the main features of the data while maintaining low error values. Additionally, all muscles exhibit r values within the moderate to strong correlation range, thereby avoiding the risk of overfitting.

In addition, we evaluated the ability of the NMF synergy-assisted EMG-driven NMSK model to estimate joint moments. The results showed that changes in the number of synergies did not have a significant effect on the accuracy of joint moment estimation (see Supplementary Figures S10–S13). However, the omission of specific muscles led to significant differences in joint moment estimation (see Supplementary Tables S6–S9). Specifically, the omission of the PD significantly affected the estimation of both arm adduction–abduction and flexion–extension moments. The absence of the TB substantially impacted the estimation of arm adduction–abduction moments as well as elbow flexion–extension moments. Meanwhile, the exclusion of the IF notably influenced the estimation of arm internal–external rotation moments. Notably, the joint moment estimations obtained from the NMF synergy-assisted EMG-driven NMSK model were consistent with those obtained from the EMG-driven NMSK model with all muscles.

## 4 Discussion

In this study, we developed an EMG-driven NMSK model tailored for the DP technique in cross-country sit-skiing. By comparing the model predictions with experimental data, we validated its ability to accurately calculate the coupled joint moments of the shoulder and elbow during the DP motion. Furthermore, based on this model, we established a synergy-assisted EMG-driven NMSK model to predict the activation patterns of missing muscles during upper limb movements and systematically investigated the impact of synergy number on prediction performance. Regarding synergy analysis methodology, we comparatively evaluated four decomposition approaches: NMF, PCA, ICA, and FA. The results demonstrated that the NMF method significantly outperformed other methods, and when the synergy number was set to 5, the synergy-assisted EMG-driven NMSK model could accurately predict the activation patterns of any missing muscle among the 10 muscles, with low error and moderate-to-strong correlation.

By collecting surface EMG data from 10 upper limb muscles, we constructed and calibrated individualized EMG-driven NMSK models for each participant (see Figure 4). The results demonstrated that the model could accurately calculate shoulder adduction-abduction moment, shoulder flexion-extension moment, and elbow flexion-extension moment. However, the model exhibited limited accuracy in predicting shoulder internal-external rotation moment. This limitation may be attributed to several factors. First, the study did not fully capture the activation signals of all muscles involved in shoulder internal-external rotation, particularly the deep muscles (e.g., teres minor). Second, shoulder internal-external rotation involves multifunctional muscles such as the IF, TMj, PM, and LD, which may prioritize optimizing moments for other DOFs, thereby reducing their contribution to internal-external rotation moment. Additionally, the potential roles of the BB and TB in shoulder internal-external rotation moment were not considered in the model, which may further limit its prediction accuracy for this DOF.

Based on the above model, this study established a synergy-assisted EMG-driven NMSK model to evaluate upper limb muscle activation patterns during the DP motion in cross-country sit-skiing. The study specifically examined how four synergy analysis methods (NMF, PCA, ICA, FA) influence the model’s muscle activation prediction accuracy. Since synergy analysis capability is closely related to the choice of synergy number, systematically evaluating the impact of synergy number on model prediction performance is essential. The results demonstrated that the NMF method consistently achieved optimal muscle activation prediction performance across all tested synergy numbers (Figures 5, 6). This superiority originates from its unique algorithmic properties: by enforcing non-negativity constraints on both basis vectors and activation coefficients, NMF strictly adheres to the physiological characteristics of muscle activation, effectively preventing non-physiological negative values and thereby significantly enhancing the physiological plausibility and interpretability of the results. Moreover, the NMF method exhibits exceptional noise robustness and can accurately identify muscle synergy patterns without imposing strict distributional assumptions (Tresch et al., 2006; Ebied et al., 2018; Zhao et al., 2022). This approach has been widely adopted in sports and upper limb movement analysis, demonstrating reliable predictive performance (Baifa et al., 2021; Wang et al., 2021). Notably, although Tahmid et al.'s study did not conduct systematic comparisons with other synergy methods, their application of NMF for upper limb muscle prediction also yielded excellent results (Tahmid et al., 2024).

Building upon these findings, this study conducted a systematic discussion on the predictive performance of the NMF synergy-assisted EMG-NMSK model. The results showed that when the synergy number was set to 5, the model could predict the activation of any missing muscle among the 10 muscles with low error and moderate-to-strong correlation (Figure 8; Supplementary Table S4). This finding is consistent with the results of Ao et al., who reported optimal prediction performance with a synergy number of 5 or 6 in lower limb gait studies (Ao et al., 2020). The model exhibited high accuracy in predicting shoulder muscle activation patterns, which typically have single-peaked and less complex activation profiles. However, the prediction accuracy for elbow muscles was relatively lower. For example, the BB had an r of 0.62 ± 0.28 and an RMSE of 0.26 ± 0.12, while the BRD had an r of 0.38 ± 0.36 and an RMSE of 0.29 ± 0.13. These results are comparable to those reported by Tahmid et al. in studies of five-degree-of-freedom shoulder-elbow movements (Tahmid et al., 2024). The lower accuracy may be due to the model including only three functional elbow muscles, which could lead to compensatory contributions from other muscles to satisfy the experimental elbow flexion-extension moment.

When the synergy number was set to 5, the model achieved an optimal balance between reconstruction capability and generalization ability, effectively capturing the main features of muscle activation while avoiding overfitting and performance degradation. The reasons for this phenomenon are as follows: First, as the synergy number increases, the model’s ability to reconstruct the original muscle activation matrix improves. However, when the synergy number exceeds a certain threshold, the reconstruction capability of NMF saturates (Santuz et al., 2020). At the same time, the dimensionality reduction capability of synergy analysis diminishes, leading to a significant increase in the number of unknown parameters during optimization. This increase in parameters makes the model more prone to fitting noise or irrelevant details, thereby reducing its generalization ability. This phenomenon explains why RMSE reached its lowest value when the synergy number was 5 and increased when the synergy number was further increased to 6 or 7. Second, the synergy-assisted method uses the dimensionality-reduced activation matrix $H_{m}$ derived from known muscle activations as the motor primitive curve ( $H_{m+x}$ ) for the 10-muscle activation matrix. To further validate the impact of synergy number on model prediction performance, we calculated the RMSE and r between $H_{m}$ and $H_{m+x}$ (Supplementary Table S5). The results showed that when the synergy number increased to 6 or 7, the r of the motor primitive curve significantly decreased (to 0.73 ± 0.33 and 0.72 ± 0.32, respectively, p < 0.05), while the RMSE significantly increased (to 0.12 ± 0.07 and 0.11 ± 0.06, respectively; p < 0.05). This indicates that although increasing the synergy number can restore more activation curve information, the error in $H_{m}$ increases, leading to poorer prediction performance for missing muscle activations.

Our study utilized a NMF synergy-assisted EMG-driven NMSK model to evaluate joint moment estimation capabilities when predicting the missing of individual muscles. The results indicated that variations in the number of synergies did not significantly affect moment estimation accuracy. However, among the muscles examined, the PD, TB, and IF demonstrated significantly greater impacts on the precision of moment predictions compared to other muscles (Supplementary Tables S3–S6). Specifically, PD, with its substantial moment arms for shoulder extension and abduction, plays a critical role in generating arm adduction-abduction and flexion-extension moments (Hik and Ackland, 2019). TB, characterized by a large maximum isometric force and an important moment arm for elbow extension, significantly influences elbow flexion-extension moments (Itoi et al., 2008). IF, a key component of the rotator cuff, is essential for shoulder stability and internal-external rotation (Hik and Ackland, 2019). These findings align with prior studies by Rabbi et al. and Steel et al., which emphasized that in scenarios involving fewer muscle combinations, dominant synergy muscles or those with greater maximum isometric force substantially affect predictive performance (Steele et al., 2013; Rabbi et al., 2022). Notably, while Ao et al. and Rabbi et al. focused on lower-limb movement studies, their conclusion that selecting muscles with significant joint moment contributions optimizes the accuracy of synergy-based extrapolation for predicting remaining muscle activations and joint forces using limited muscle data aligns with our findings (Ao and Fregly, 2024; Rabbi et al., 2024). Our results provide a foundation for future investigations into predicting the activation patterns of remaining muscles during DP movements with reduced muscle sets, highlighting the critical roles of PD, TB, and IF.

Despite the promising results, this study has certain limitations. First, the model did not incorporate the activation patterns of deep muscles in the shoulder and elbow joints, which may have contributed to moment calculation errors. Second, the BUET model used in this study constrained the sternoclavicular and acromioclavicular joints, without considering their potential influence on the results. Third, the small sample size (n = 3) limits the generalizability of the findings. To address these limitations, future studies will: (1) optimize the model structure to include deep muscle activations; (2) relax joint constraints in the BUET model to investigate their biomechanical effects; (3) explore the feasibility of predicting a larger number of missing muscle activations using fewer known activations; and (4) validate the model with a larger cohort to establish its generalizability.

## 5 Conclusion

This study developed an EMG-driven NMSK model tailored for the DP technique in cross-country sit-skiing, capable of accurately calculating the joint moments of the shoulder and elbow during coupled movements. Furthermore, to address the issue of missing muscle data in the collected dataset, a synergy-assisted EMG-driven NMSK model was further developed. The results suggest that NMF synergy-assisted method with the synergy number to 5 allows the model to reasonably predict the activation patterns of any missing muscles among the 10 muscles. However, given the limited number of participants and the observed accuracy levels, these findings should be interpreted with caution.

## Data Availability

The original contributions presented in the study are included in the article/Supplementary Material, further inquiries can be directed to the corresponding authors.
