# Supplementary material for: Assessment of synergy-assisted EMG-driven NMSK model for upper limb muscle activation prediction in cross-country sit-skiing double poling
Source: Front Bioeng Biotechnol. 2025 Aug 18;13:1585127. doi: 10.3389/fbioe.2025.1585127 (PMC12399978; doi:10.3389/fbioe.2025.1585127)
Supplement: Supplementary file 1 [file Supplementaryfile1.docx]

Supplementary Material

# Synergy-Assisted Method

To screen the optimal synergy-assisted analysis method, this study employs four approaches—Non-Negative Matrix Factorization (NMF), Principal Component Analysis (PCA), Independent Component Analysis (ICA), and Factor Analysis (FA)—to predict single-missing upper limb muscle activations (as shown in Figure 1).NMF utilizes nonlinear optimization algorithms to iteratively obtain solutions under non-negativity constraints, with potential non-unique solutions (details in section 2.5); PCA derives unique analytical solutions based on linear algebra principles under orthogonality constraints, optimally explaining data variance by identifying internal structures; ICA separates mixed signals by maximizing non-Gaussianity according to statistical independence assumptions; FA constructs latent variable models that reveal underlying relationships among observed variables through covariance matrix decomposition. The detailed computational procedures are as follows:

$a_{m}=\left\{ \begin{aligned} &W_{m}H_{m}+\mu_{m}&\left( \mathrm{PCA} \right) \\ &W_{m}H_{m}+\mu_{m}&\left( \mathrm{ICA} \right) \\ &W_{m}H_{m}+\psi_{m}+\mu_{m}&\left( \mathrm{FA} \right) \end{aligned} \right.$

where $a_{m}$ denotes the measured muscle activation matrix ($m\times n$, $m$= 9 measured muscles and $n$= 100 time points); $W_{m}$ represents the relative weights of individual muscles ($m\times l$), where $l$ denotes the number of synergies; and $H_{m}$ represents the time-dependent coefficients ($l\times n$); for PCA, ICA, and FA, $\mu_{m}$ denotes the average muscle activations across time points in $a_{m}$ ($m\times$ 1); for FA, $\psi_{m}$ denotes the unique variance (diagonal matrix) accounting for muscle-specific variability not explained by the common factors ($m\times n$). PCA was implemented using MATLAB's "pca" function, while ICA and FA were performed via the "fastica" and "factoran" functions, respectively.

The unmeasured muscle activation $a_{x}$ was derived from the extracted synergy time-dependent coefficients $H_{m}$ using the following formulation:

$a_{x}=\left\{ \begin{aligned} &W_{x}H_{m}+\mu_{x}&\left( \mathrm{PCA} \right) \\ &W_{x}H_{m}+\mu_{x}&\left( \mathrm{ICA} \right) \\ &W_{x}H_{m}+\psi_{x}+\mu_{x}&\left( \mathrm{FA} \right) \end{aligned} \right.$

where $a_{x}$ denotes unmeasured muscle activation matrix (1 $\times n$); $W_{x}$ represents the synergy weight matrix for unmeasured muscle (1 $\times l$); $\mu_{x}$ denotes the average value of the unmeasured muscle activation (1 $\times$ 1); $\psi_{x}$ indicates the unique variance component of the unmeasured muscle activation (1 $\times n$). For PCA, ICA, and FA, the parameters $W_{x}$, $\mu_{x}$, and $\psi_{x}$ were obtained through optimization iterations by tracking the experimental joint moments. The optimization process initialized these parameters with random values bounded between 0 and 1, with $W_{x}$ and $\psi_{x}$ were allowed to vary without bounds, $\mu_{x}$ was constrained to range between 0 and 1, and $a_{x}$ were restricted to a range between 0 and 1.

#

# EMG-Driven NMSK Model

## Hill-type model


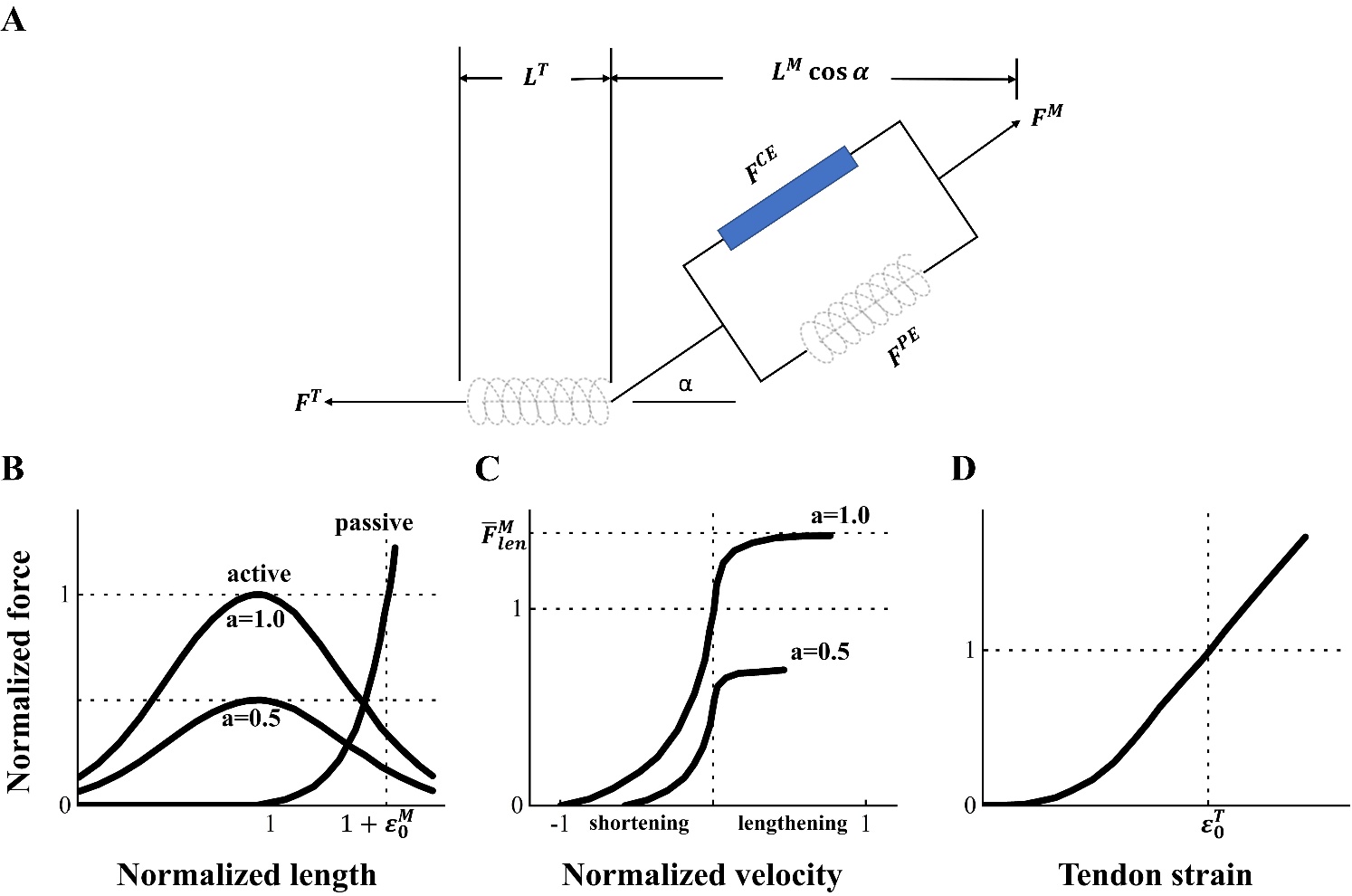


**Supplementary Figure 1.** Muscle and tendon modeling components. **A** Hill-type model. **B** Active muscle force-activation-length-velocity relationship. **C** Passive muscle force-length relationship. **D** Tendon length-tendon force relationship.

## Joint angles


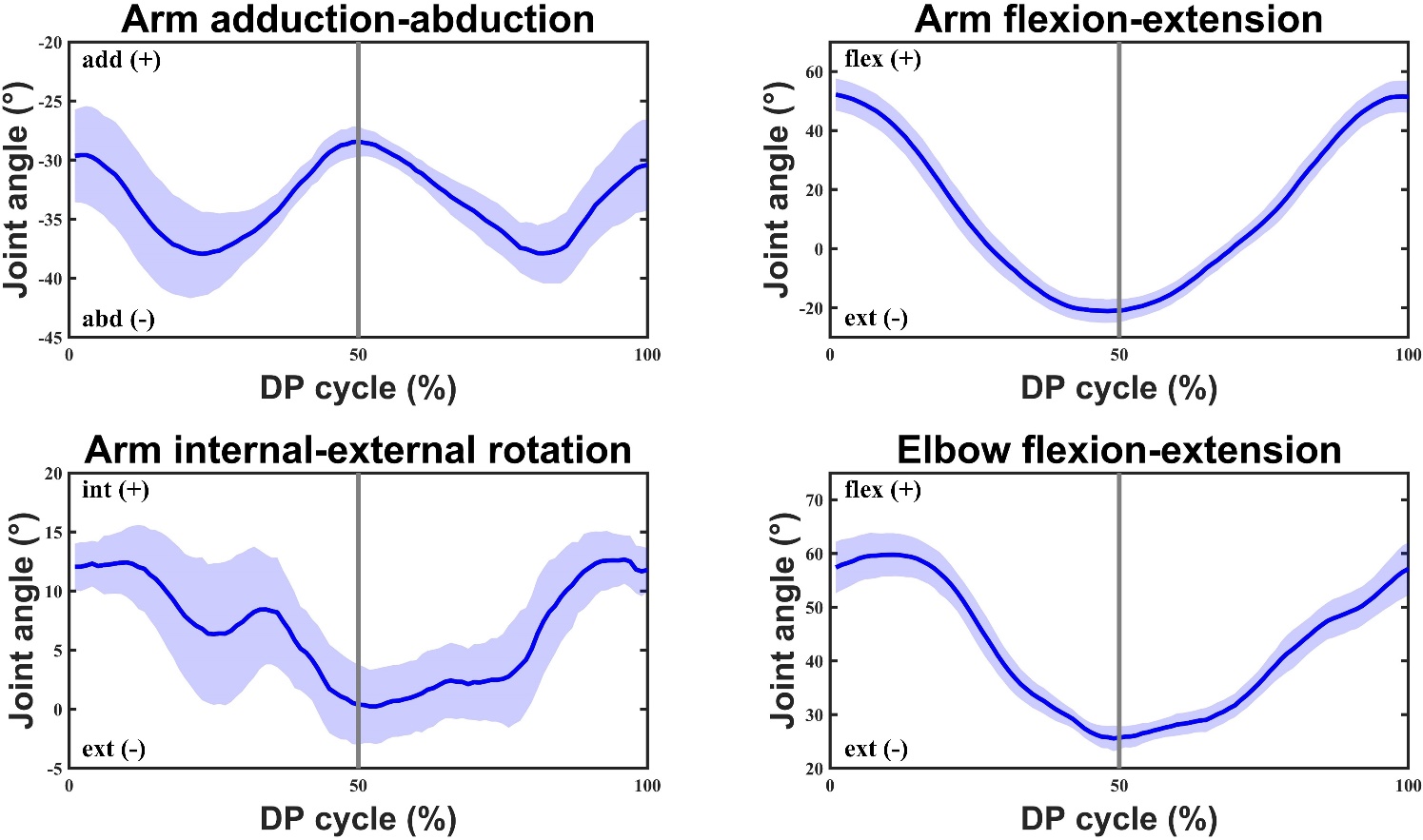


**Supplementary Figure 2.** The average joint angles for four degrees of freedom at the shoulder and elbow joints were simulated using inverse kinematics for three participants. Positive values represent arm adduction, flexion, internal rotation, and elbow flexion.

# Methodological Choices for Synergy-Assisted

## Muscle activation prediction (PCA)


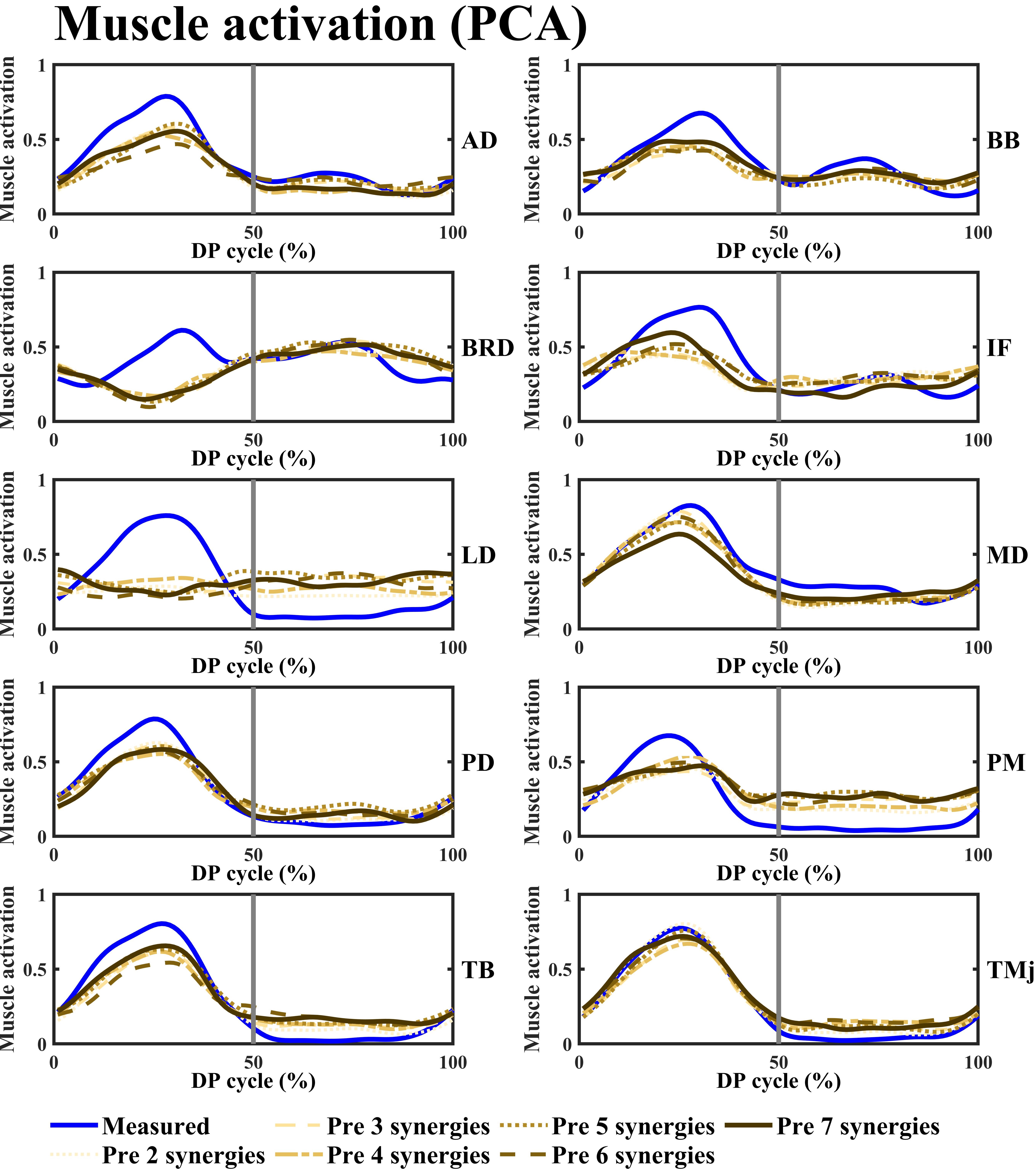


**Supplementary Figure 3.** The variation curves of the predicted average missing muscle activation and the experimentally measured average muscle activation under different synergy number conditions were plotted. The blue curve represents the experimentally measured values, while the yellow curve corresponds to the predicted values calculated using the PCA synergy-assisted EMG-driven NMSK model. The 0%-50% of the cycle corresponds to the poling phase (PP) of the double poling (DP), while the 50%-100% corresponds to the recovery phase (RP). Muscle abbreviations: AD, anterior deltoid; BB, biceps brachii; BRD, brachioradialis; IF, infraspinatus; LD, latissimus dorsi; MD, middle deltoid; PD, posterior deltoid; PM, pectoralis major; TB, triceps brachii; TMj, teres major.


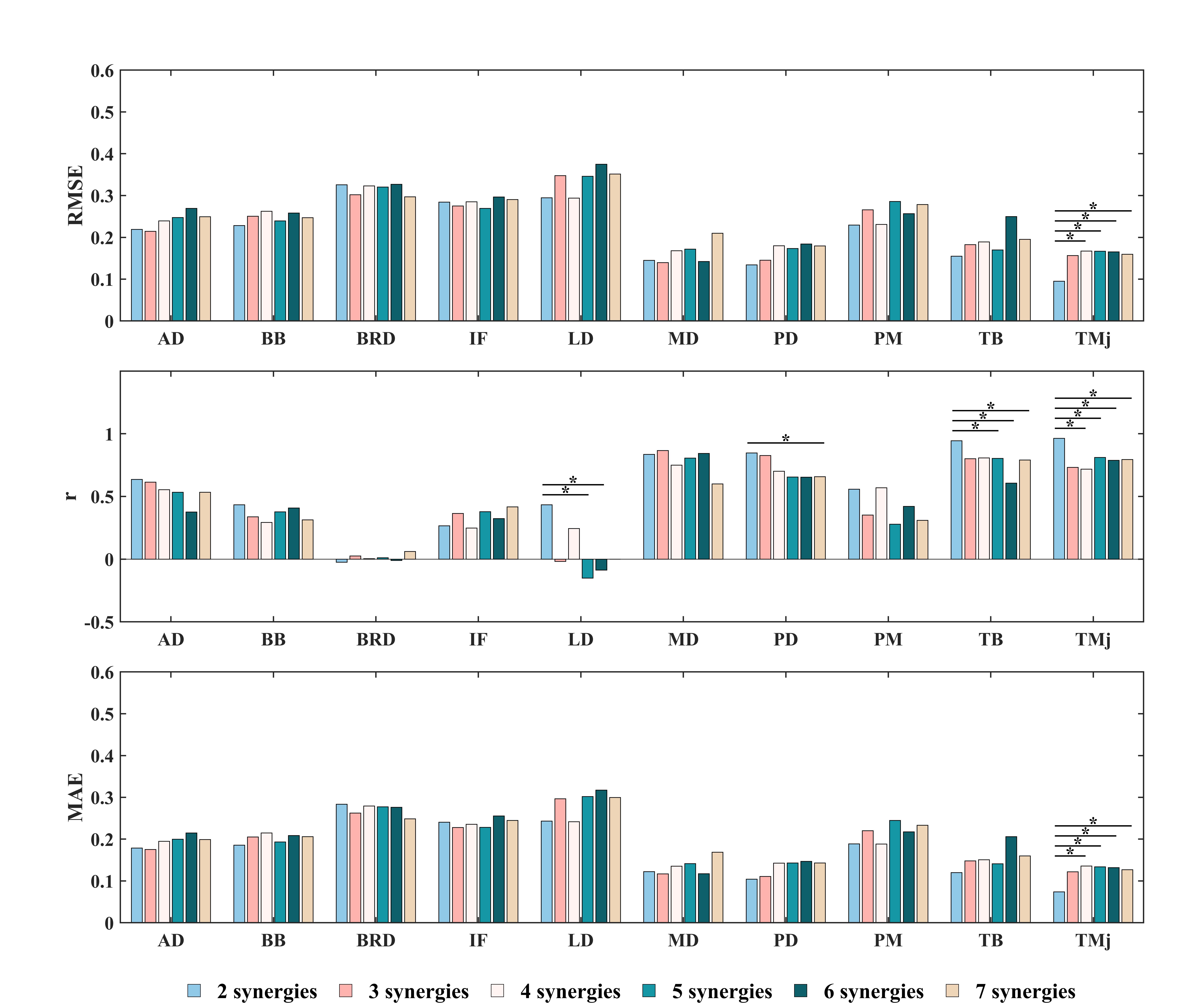


**Supplementary Figure 4.** The RMSE, $r$, and MAE between the predicted missing muscle activations by the PCA synergy-assisted EMG-driven NMSK model under different synergy numbers and the experimental measurements. * indicates significant differences between groups ($p$< 0.05). Muscle abbreviations: AD, anterior deltoid; BB, biceps brachii; BRD, brachioradialis; IF, infraspinatus; LD, latissimus dorsi; MD, middle deltoid; PD, posterior deltoid; PM, pectoralis major; TB, triceps brachii; TMj, teres major.

## Muscle activation prediction (ICA)


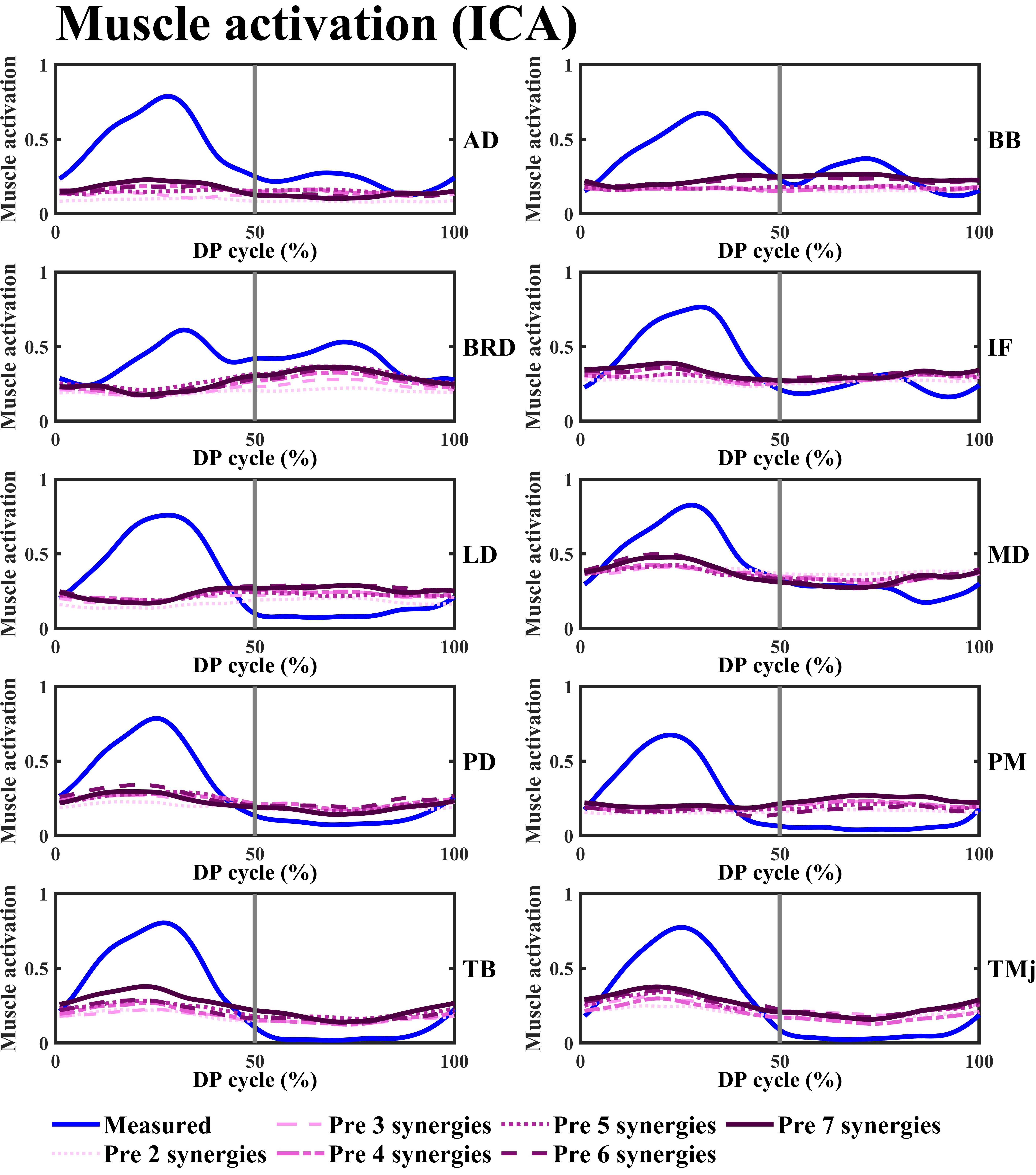


**Supplementary Figure 5.** The variation curves of the predicted average missing muscle activation and the experimentally measured average muscle activation under different synergy number conditions were plotted. The blue curve represents the experimentally measured values, while the purple curve corresponds to the predicted values calculated using the ICA synergy-assisted EMG-driven NMSK model. The 0%-50% of the cycle corresponds to the poling phase (PP) of the double poling (DP), while the 50%-100% corresponds to the recovery phase (RP). Muscle abbreviations: AD, anterior deltoid; BB, biceps brachii; BRD, brachioradialis; IF, infraspinatus; LD, latissimus dorsi; MD, middle deltoid; PD, posterior deltoid; PM, pectoralis major; TB, triceps brachii; TMj, teres major.


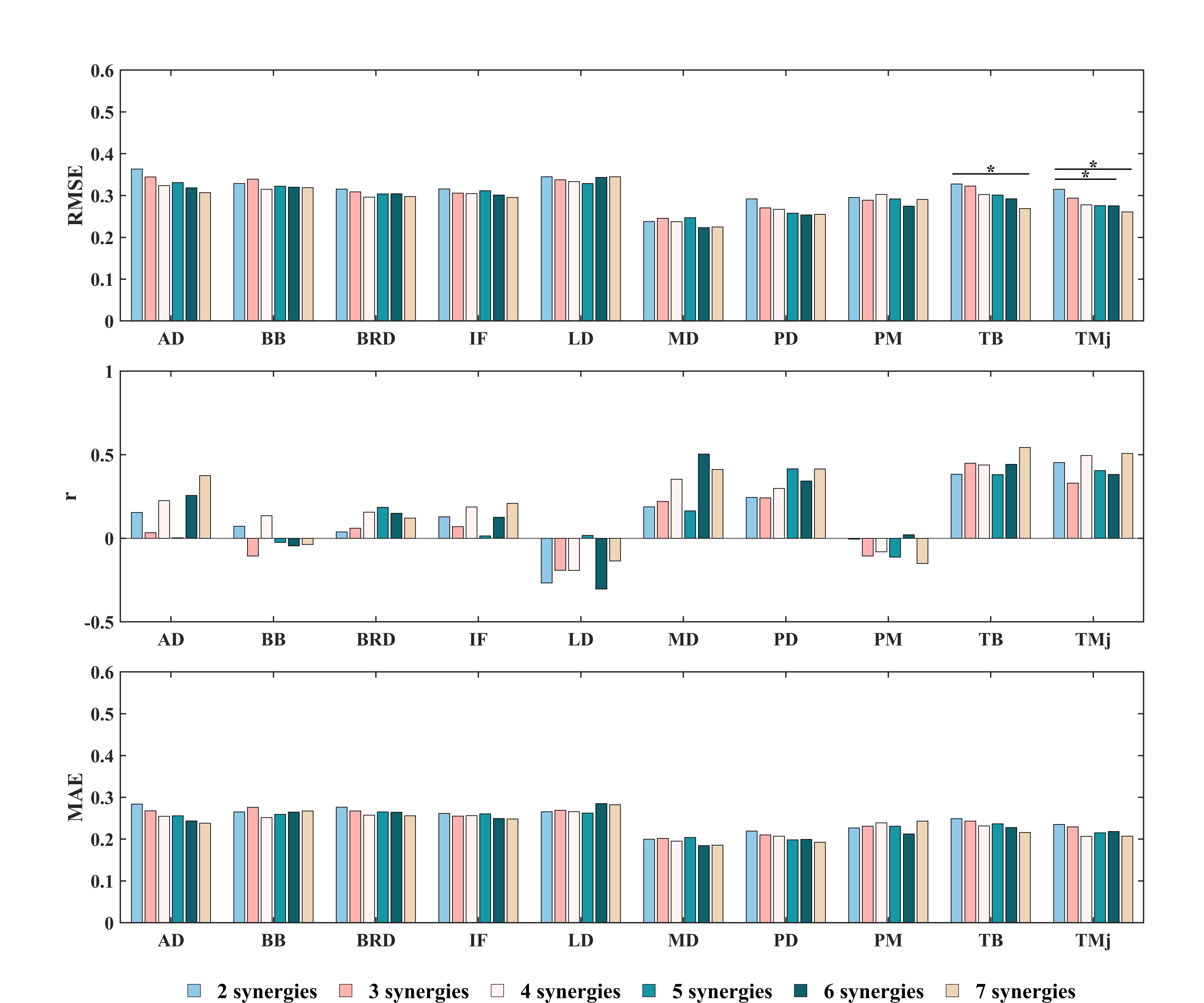


**Supplementary Figure 6.** The RMSE, $r$, and MAE between the predicted missing muscle activations by the ICA synergy-assisted EMG-driven NMSK model under different synergy numbers and the experimental measurements. * indicates significant differences between groups ($p$< 0.05). Muscle abbreviations: AD, anterior deltoid; BB, biceps brachii; BRD, brachioradialis; IF, infraspinatus; LD, latissimus dorsi; MD, middle deltoid; PD, posterior deltoid; PM, pectoralis major; TB, triceps brachii; TMj, teres major.

## Muscle activation prediction (FA)


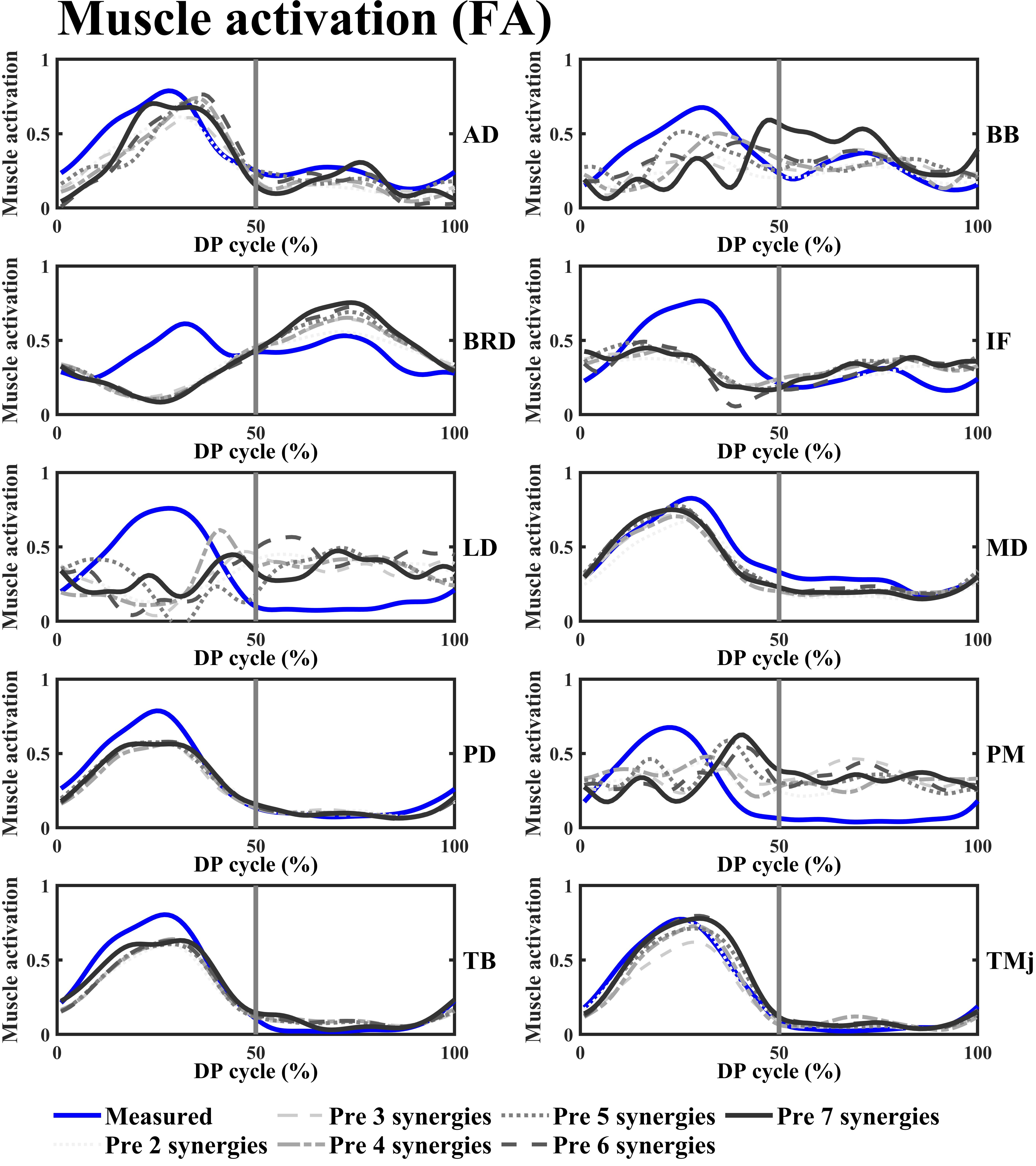


**Supplementary Figure 7.** The variation curves of the predicted average missing muscle activation and the experimentally measured average muscle activation under different synergy number conditions were plotted. The blue curve represents the experimentally measured values, while the gray curve corresponds to the predicted values calculated using the FA synergy-assisted EMG-driven NMSK model. The 0%-50% of the cycle corresponds to the poling phase (PP) of the double poling (DP), while the 50%-100% corresponds to the recovery phase (RP). Muscle abbreviations: AD, anterior deltoid; BB, biceps brachii; BRD, brachioradialis; IF, infraspinatus; LD, latissimus dorsi; MD, middle deltoid; PD, posterior deltoid; PM, pectoralis major; TB, triceps brachii; TMj, teres major.


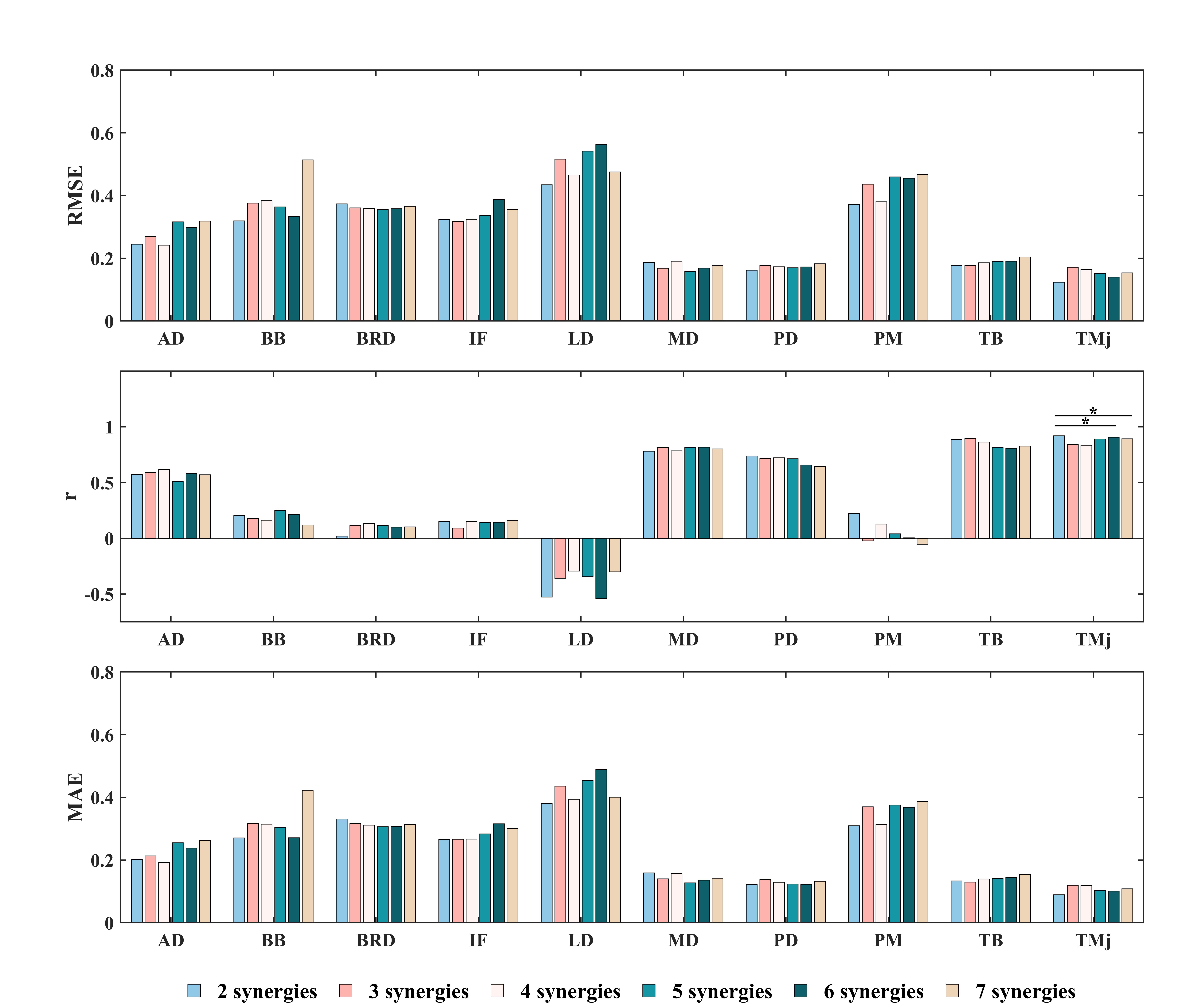


**Supplementary Figure 8.** The RMSE, $r$, and MAE between the predicted missing muscle activations by the FA synergy-assisted EMG-driven NMSK model under different synergy numbers and the experimental measurements. * indicates significant differences between groups ($p$< 0.05). Muscle abbreviations: AD, anterior deltoid; BB, biceps brachii; BRD, brachioradialis; IF, infraspinatus; LD, latissimus dorsi; MD, middle deltoid; PD, posterior deltoid; PM, pectoralis major; TB, triceps brachii; TMj, teres major.

## Comprehensive evaluation of four synergy extraction methods


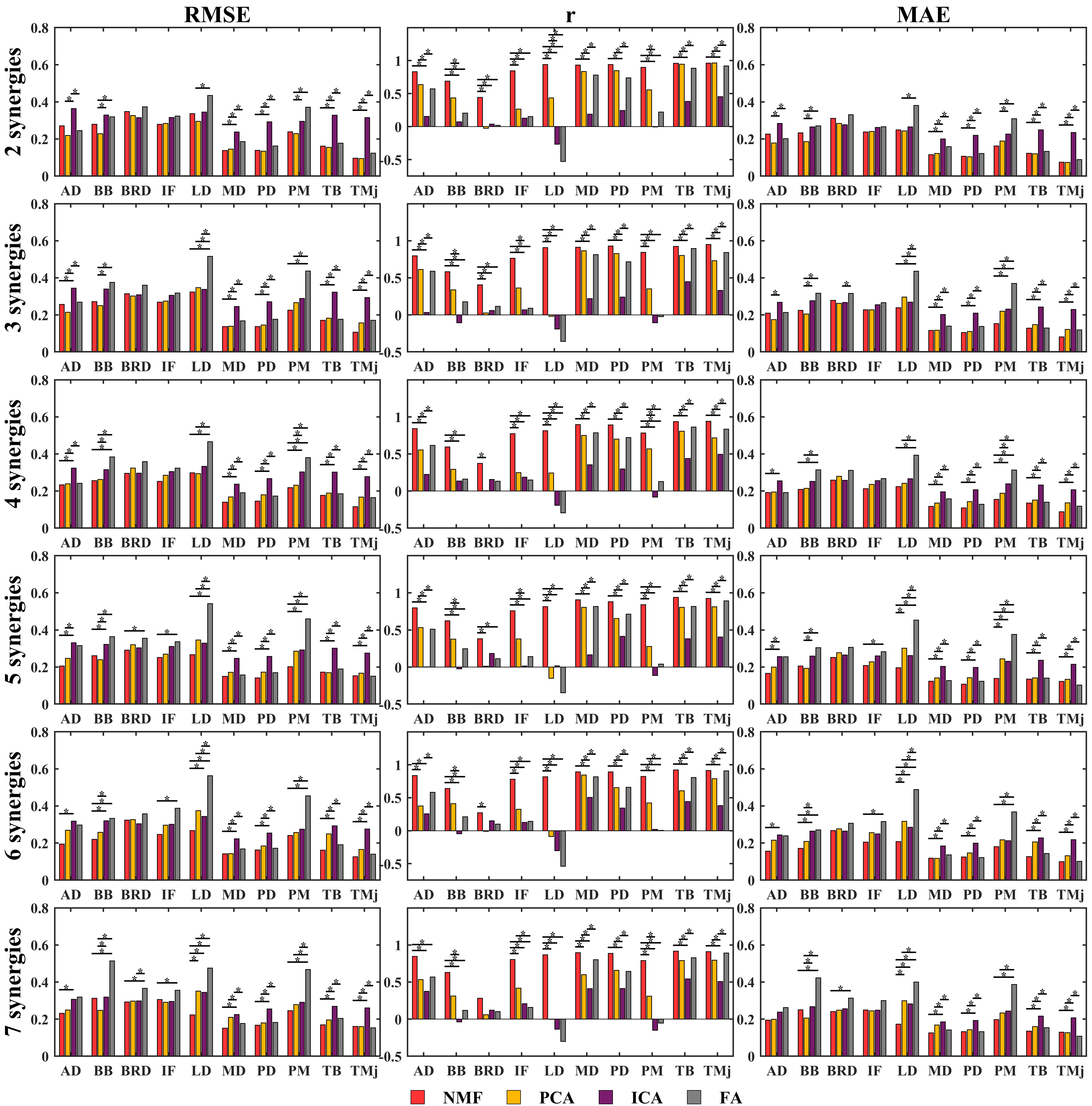


**Supplementary Figure 9.** The RMSE, $r$, and MAE between the predicted missing muscle activations by the synergy-assisted EMG-driven NMSK model using different synergistic assistance methods under varying synergy numbers and the experimental measurements. Red represents NMF, yellow represents PCA, purple represents ICA, gray represents FA. * indicates significant differences between groups ($p$< 0.05). Muscle abbreviations: AD, anterior deltoid; BB, biceps brachii; BRD, brachioradialis; IF, infraspinatus; LD, latissimus dorsi; MD, middle deltoid; PD, posterior deltoid; PM, pectoralis major; TB, triceps brachii; TMj, teres major.

# Joint moments (NMF)


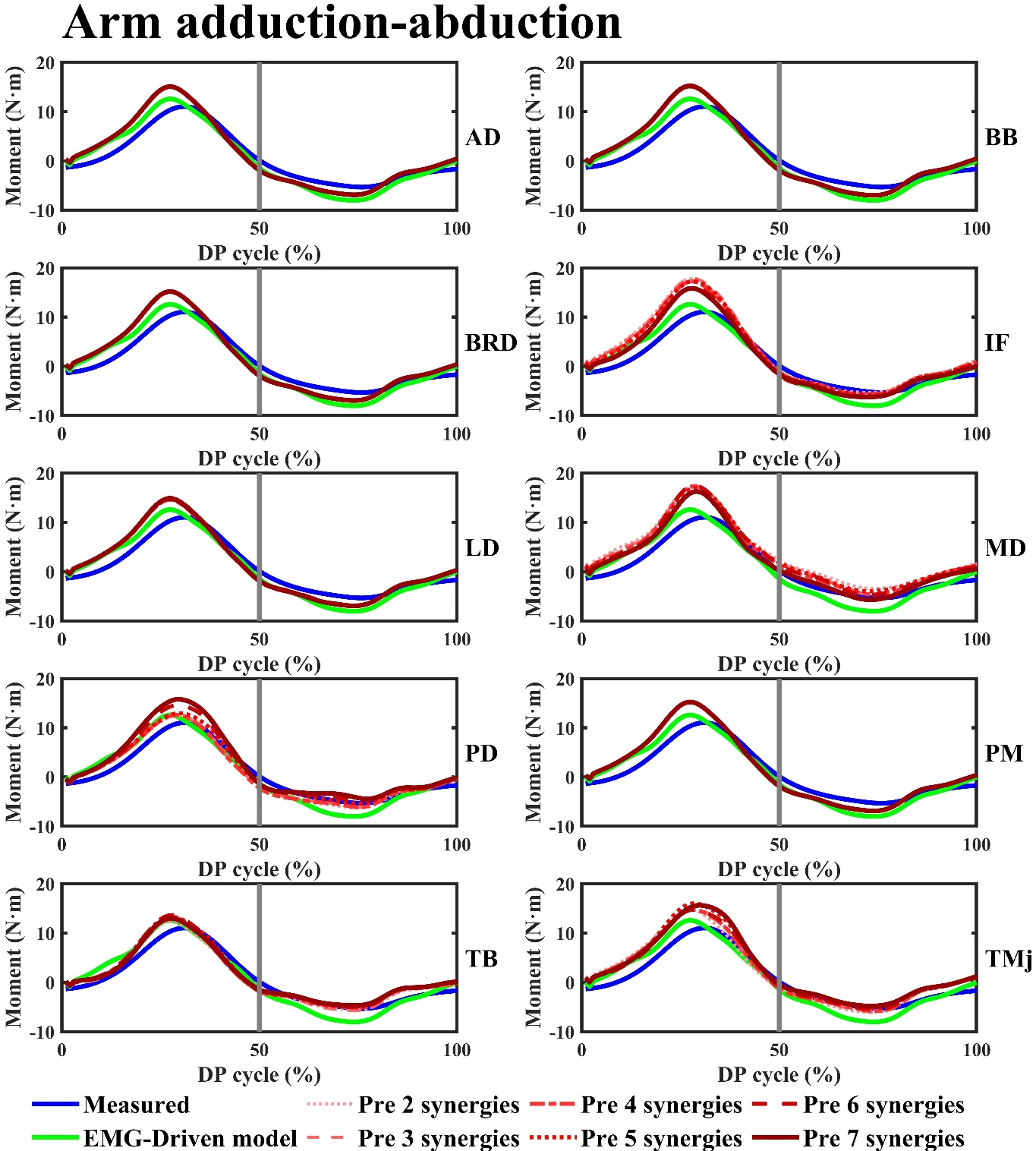


**Supplementary Figure 10.** The arm adduction–abduction joint moments estimated by the NMF synergy-assisted EMG-driven NMSK model (red curve) during the prediction of a specific muscle, using varying numbers of synergists, were compared with those obtained from the EMG-driven NMSK model with all muscles included (green curve) and from inverse dynamics analysis (blue curve). The results indicate that the omission of individual muscles did not substantially impact the accuracy of joint moment estimation. The 0%-50% of the cycle corresponds to the poling phase (PP) of the double poling (DP), while the 50%-100% corresponds to the recovery phase (RP). Muscle abbreviations: AD, anterior deltoid; BB, biceps brachii; BRD, brachioradialis; IF, infraspinatus; LD, latissimus dorsi; MD, middle deltoid; PD, posterior deltoid; PM, pectoralis major; TB, triceps brachii; TMj, teres major.


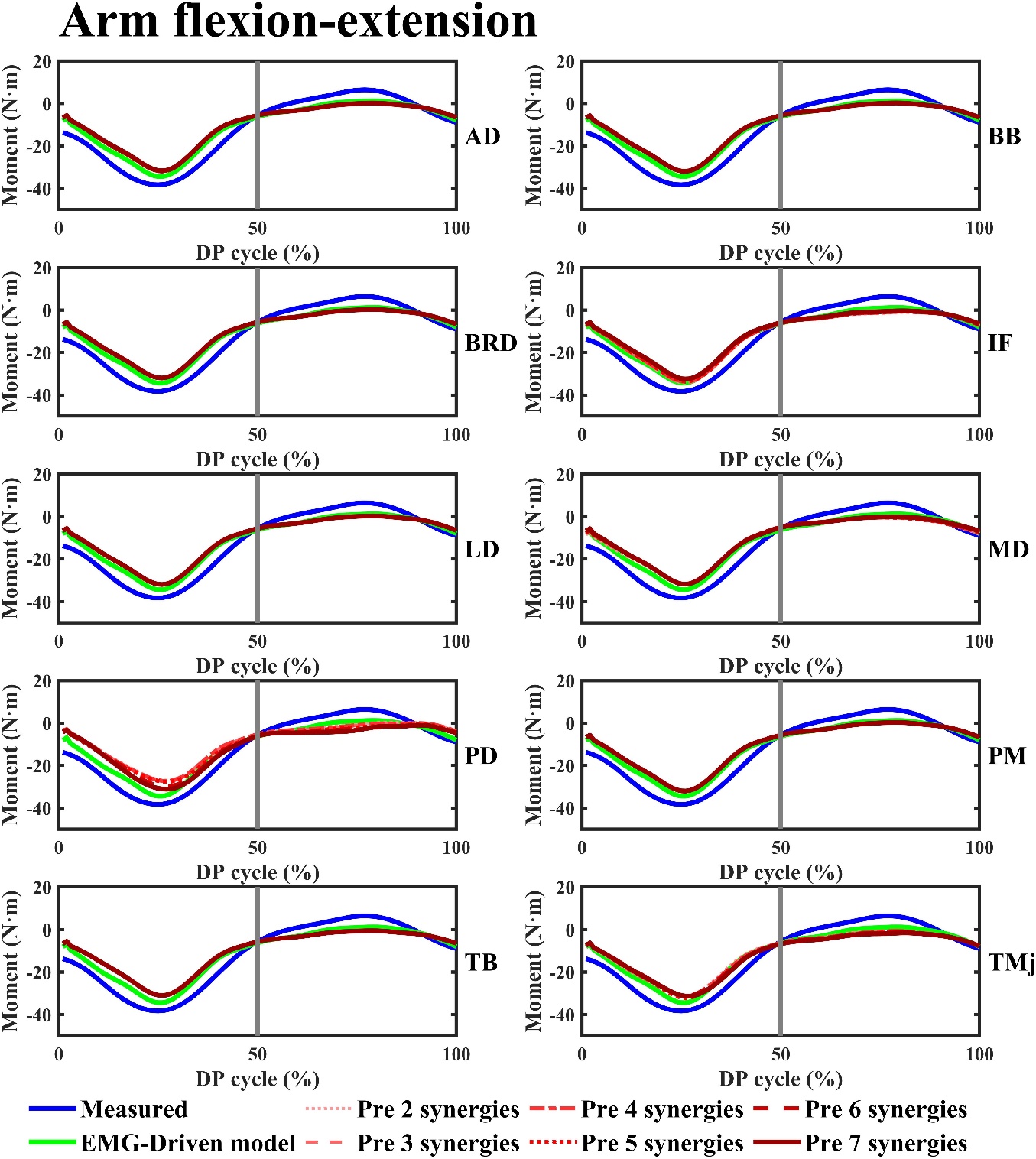


**Supplementary Figure 11.** The arm flexion–extension joint moments estimated by the NMF synergy-assisted EMG-driven NMSK model (red curve) during the prediction of a specific muscle, using varying numbers of synergists, were compared with those obtained from the EMG-driven NMSK model with all muscles included (green curve) and from inverse dynamics analysis (blue curve). The results indicate that the omission of individual muscles did not substantially impact the accuracy of joint moment estimation. The 0%-50% of the cycle corresponds to the poling phase (PP) of the double poling (DP), while the 50%-100% corresponds to the recovery phase (RP). Muscle abbreviations: AD, anterior deltoid; BB, biceps brachii; BRD, brachioradialis; IF, infraspinatus; LD, latissimus dorsi; MD, middle deltoid; PD, posterior deltoid; PM, pectoralis major; TB, triceps brachii; TMj, teres major.


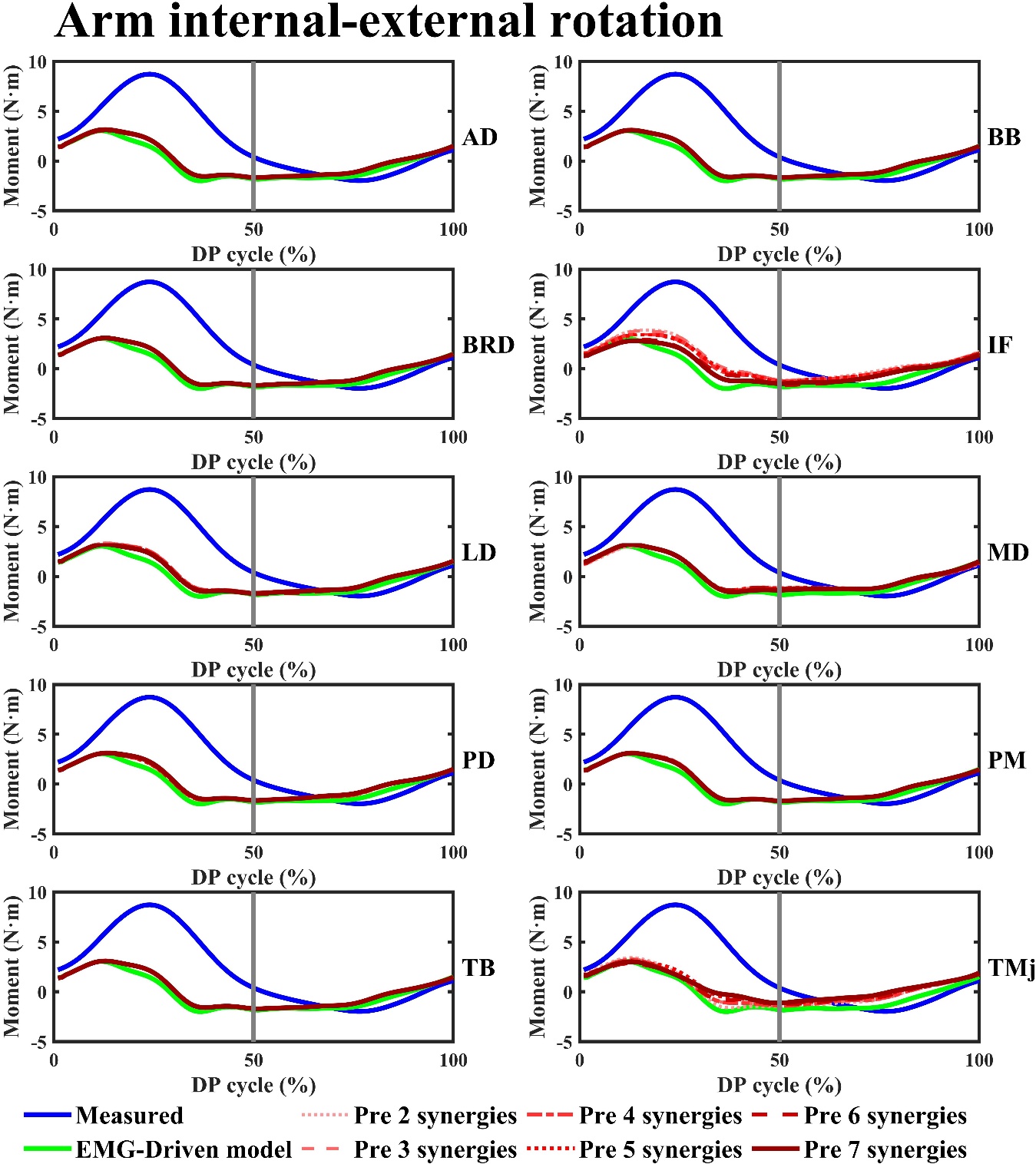


**Supplementary Figure 12.** The arm internal-external rotation joint moments estimated by the NMF synergy-assisted EMG-driven NMSK model (red curve) during the prediction of a specific muscle, using varying numbers of synergists, were compared with those obtained from the EMG-driven NMSK model with all muscles included (green curve) and from inverse dynamics analysis (blue curve). The results indicate that the omission of individual muscles did not substantially impact the accuracy of joint moment estimation. The 0%-50% of the cycle corresponds to the poling phase (PP) of the double poling (DP), while the 50%-100% corresponds to the recovery phase (RP). Muscle abbreviations: AD, anterior deltoid; BB, biceps brachii; BRD, brachioradialis; IF, infraspinatus; LD, latissimus dorsi; MD, middle deltoid; PD, posterior deltoid; PM, pectoralis major; TB, triceps brachii; TMj, teres major.


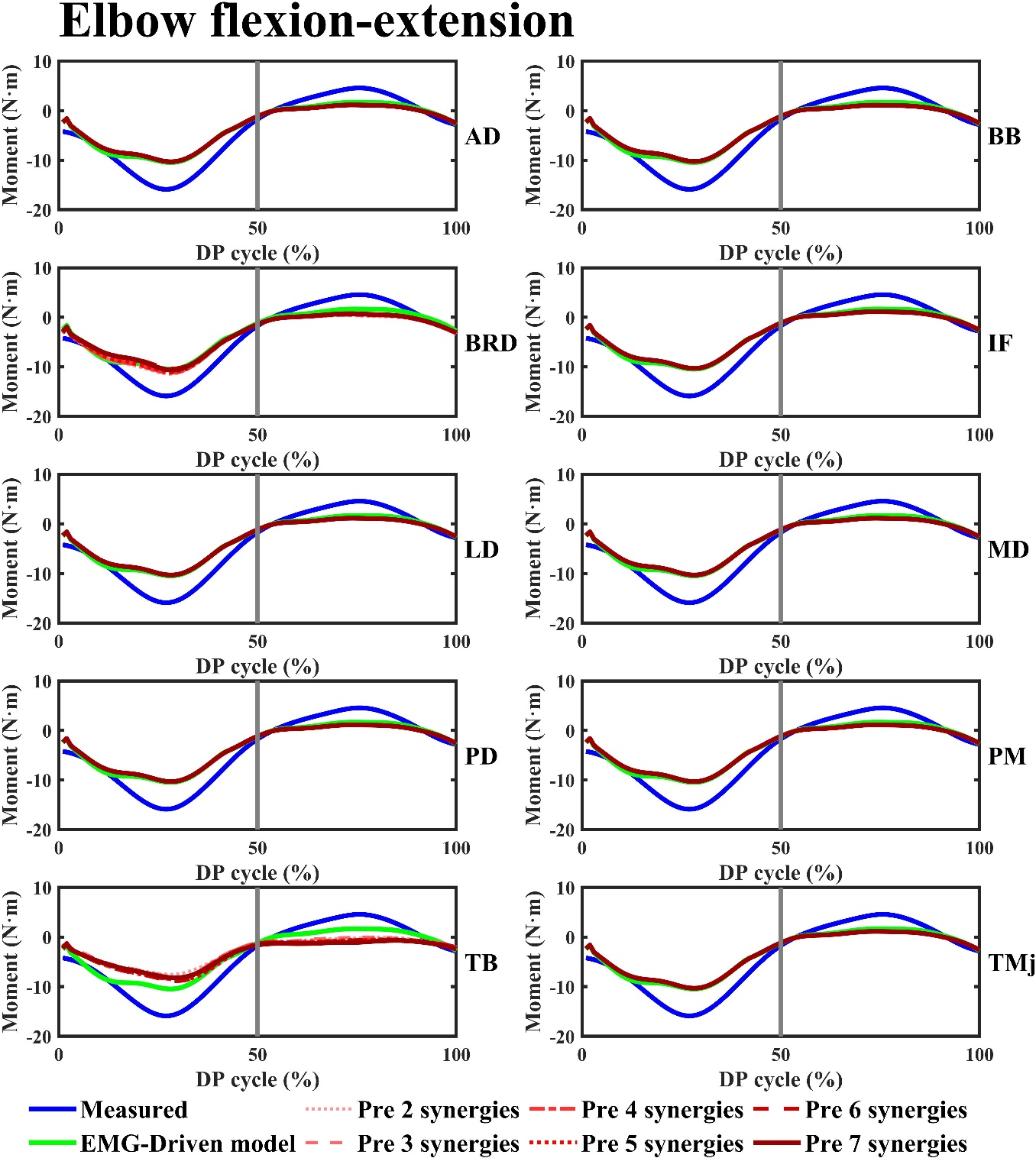


**Supplementary Figure 13.** The elbow flexion-extension joint moments estimated by the NMF synergy-assisted EMG-driven NMSK model (red curve) during the prediction of a specific muscle, using varying numbers of synergists, were compared with those obtained from the EMG-driven NMSK model with all muscles included (green curve) and from inverse dynamics analysis (blue curve). The results indicate that the omission of individual muscles did not substantially impact the accuracy of joint moment estimation. The 0%-50% of the cycle corresponds to the poling phase (PP) of the double poling (DP), while the 50%-100% corresponds to the recovery phase (RP). Muscle abbreviations: AD, anterior deltoid; BB, biceps brachii; BRD, brachioradialis; IF, infraspinatus; LD, latissimus dorsi; MD, middle deltoid; PD, posterior deltoid; PM, pectoralis major; TB, triceps brachii; TMj, teres major.

# Supplementary Tables

Table 1 The errors between the muscle activation predicted by the PCA synergy-assisted EMG-driven NMSK model and the experimental measurements different numbers of synergies were evaluated using RMSE, $r$, and MAE.

| Number of synergies |  | Muscle | | | | | | | | | |
| --- | --- | --- | --- | --- | --- | --- | --- | --- | --- | --- | --- |
|  |  | AD | BB | BRD | IF | LD | MD | PD | PM | TB | TMj |
| 2 | RMSE | 0.22±0.13 | 0.23±0.11 | 0.33±0.07 | 0.28±0.11 | 0.29±0.15 | 0.15±0.07 | 0.13±0.07 | 0.23±0.16 | 0.15±0.07 | 0.09±0.03 |
|  | $r$ | 0.64±0.56 | 0.43±0.52 | -0.03±0.43 | 0.27±0.73 | 0.43±0.81 | 0.84±0.30 | 0.85±0.39 | 0.56±0.68 | 0.94±0.06 | 0.96±0.02 |
|  | MAE | 0.18±0.11 | 0.19±0.10 | 0.28±0.08 | 0.24±0.10 | 0.24±0.15 | 0.12±0.06 | 0.10±0.05 | 0.19±0.15 | 0.12±0.05 | 0.07±0.02 |
| 3 | RMSE | 0.21±0.11 | 0.25±0.16 | 0.30±0.09 | 0.27±0.12 | 0.35±0.16 | 0.14±0.04 | 0.15±0.06 | 0.27±0.16 | 0.18±0.10 | 0.16±0.11 |
|  | $r$ | 0.61±0.58 | 0.34±0.58 | 0.03±0.42 | 0.36±0.69 | -0.02±0.86 | 0.87±0.11 | 0.83±0.29 | 0.35±0.75 | 0.80±0.46 | 0.73±0.53 |
|  | MAE | 0.18±0.09 | 0.21±0.13 | 0.26±0.08 | 0.23±0.11 | 0.30±0.17 | 0.12±0.03 | 0.11±0.05 | 0.22±0.15 | 0.15±0.09 | 0.12±0.09 |
| 4 | RMSE | 0.24±0.11 | 0.26±0.17 | 0.32±0.10 | 0.29±0.11 | 0.29±0.15 | 0.17±0.08 | 0.18±0.16 | 0.23±0.11 | 0.19±0.15 | 0.17±0.12**^a^** |
|  | $r$ | 0.55±0.52 | 0.29±0.62 | 0.00±0.35 | 0.25±0.68 | 0.24±0.73 | 0.75±0.41 | 0.70±0.54 | 0.57±0.56 | 0.81±0.42 | 0.72±0.55**^a^** |
|  | MAE | 0.19±0.10 | 0.21±0.13 | 0.28±0.09 | 0.24±0.09 | 0.24±0.15 | 0.14±0.06 | 0.14±0.12 | 0.19±0.11 | 0.15±0.12 | 0.14±0.11**^a^** |
| 5 | RMSE | 0.25±0.11 | 0.24±0.10 | 0.32±0.07 | 0.27±0.10 | 0.35±0.17 | 0.17±0.14 | 0.17±0.11 | 0.29±0.16 | 0.17±0.09 | 0.17±0.12**^a^** |
|  | $r$ | 0.53±0.60 | 0.38±0.53 | 0.01±0.45 | 0.38±0.59 | -0.15±0.82 | 0.81±0.31 | 0.65±0.55 | 0.28±0.68 | 0.80±0.40**^a^** | 0.81±0.36**^a^** |
|  | MAE | 0.20±0.10 | 0.19±0.09 | 0.28±0.07 | 0.23±0.10 | 0.30±0.16 | 0.14±0.10 | 0.14±0.11 | 0.24±0.15 | 0.14±0.08 | 0.13±0.10**^a^** |
| 6 | RMSE | 0.27±0.18 | 0.26±0.10 | 0.33±0.08 | 0.30±0.12 | 0.37±0.22 | 0.14±0.05 | 0.18±0.12 | 0.26±0.14 | 0.25±0.20 | 0.17±0.13**^a^** |
|  | $r$ | 0.38±0.60 | 0.41±0.50 | -0.01±0.36 | 0.32±0.65 | -0.09±0.79**^a^** | 0.84±0.21 | 0.65±0.53 | 0.42±0.61 | 0.61±0.62**^a^** | 0.79±0.45**^a^** |
|  | MAE | 0.21±0.14 | 0.21±0.08 | 0.28±0.06 | 0.26±0.12 | 0.32±0.20 | 0.12±0.04 | 0.15±0.09 | 0.22±0.13 | 0.21±0.17 | 0.13±0.11**^a^** |
| 7 | RMSE | 0.25±0.13 | 0.25±0.10 | 0.30±0.09 | 0.29±0.11 | 0.35±0.19 | 0.21±0.16 | 0.18±0.12 | 0.28±0.15 | 0.20±0.09 | 0.16±0.10**^a^** |
|  | $r$ | 0.53±0.55 | 0.31±0.60 | 0.06±0.40 | 0.42±0.59 | -0.00±0.77**^a^** | 0.60±0.59 | 0.66±0.53**^a^** | 0.31±0.63 | 0.79±0.25**^a^** | 0.79±0.38**^a^** |
|  | MAE | 0.20±0.10 | 0.21±0.09 | 0.25±0.08 | 0.24±0.11 | 0.30±0.18 | 0.17±0.12 | 0.14±0.10 | 0.23±0.14 | 0.16±0.07 | 0.13±0.08**^a^** |

Note: Superscripts a, b, c, d, and e indicate significant differences compared to the number of synergies 2, 3, 4, 5, and 6, respectively. Red represents RMSE; blue represents r; purple represents MAE.

Table 2 The errors between the muscle activation predicted by the ICA synergy-assisted EMG-driven NMSK model and the experimental measurements different numbers of synergies were evaluated using RMSE, $r$, and MAE.

| Number of synergies |  | Muscle | | | | | | | | | |
| --- | --- | --- | --- | --- | --- | --- | --- | --- | --- | --- | --- |
|  |  | AD | BB | BRD | IF | LD | MD | PD | PM | TB | TMj |
| 2 | RMSE | 0.36±0.08 | 0.33±0.06 | 0.32±0.15 | 0.32±0.08 | 0.34±0.06 | 0.24±0.06 | 0.29±0.06 | 0.30±0.08 | 0.33±0.06 | 0.32±0.05 |
|  | $r$ | 0.15±0.57 | 0.07±0.54 | 0.04±0.43 | 0.13±0.50 | -0.27±0.59 | 0.19±0.56 | 0.24±0.59 | -0.01±0.64 | 0.38±0.59 | 0.45±0.47 |
|  | MAE | 0.28±0.08 | 0.27±0.05 | 0.28±0.15 | 0.26±0.08 | 0.27±0.06 | 0.20±0.04 | 0.22±0.05 | 0.23±0.09 | 0.25±0.06 | 0.24±0.06 |
| 3 | RMSE | 0.34±0.08 | 0.34±0.06 | 0.31±0.15 | 0.31±0.08 | 0.34±0.08 | 0.25±0.06 | 0.27±0.06 | 0.29±0.09 | 0.32±0.06 | 0.29±0.06 |
|  | $r$ | 0.03±0.53 | -0.11±0.52 | 0.06±0.40 | 0.07±0.56 | -0.19±0.62 | 0.22±0.60 | 0.24±0.69 | -0.11±0.71 | 0.45±0.48 | 0.33±0.60 |
|  | MAE | 0.27±0.08 | 0.28±0.06 | 0.27±0.15 | 0.26±0.08 | 0.27±0.08 | 0.20±0.05 | 0.21±0.05 | 0.23±0.09 | 0.24±0.05 | 0.23±0.06 |
| 4 | RMSE | 0.32±0.09 | 0.32±0.05 | 0.30±0.14 | 0.30±0.08 | 0.33±0.06 | 0.24±0.07 | 0.27±0.06 | 0.30±0.06 | 0.30±0.07 | 0.28±0.05 |
|  | $r$ | 0.22±0.58 | 0.14±0.49 | 0.16±0.38 | 0.19±0.53 | -0.19±0.63 | 0.35±0.47 | 0.30±0.60 | -0.08±0.67 | 0.44±0.57 | 0.50±0.54 |
|  | MAE | 0.25±0.08 | 0.25±0.04 | 0.26±0.14 | 0.26±0.07 | 0.27±0.06 | 0.20±0.06 | 0.21±0.06 | 0.24±0.07 | 0.23±0.05 | 0.21±0.05 |
| 5 | RMSE | 0.33±0.08 | 0.32±0.06 | 0.30±0.13 | 0.31±0.07 | 0.33±0.08 | 0.25±0.06 | 0.26±0.06 | 0.29±0.09 | 0.30±0.07 | 0.28±0.07 |
|  | $r$ | 0.00±0.54 | -0.02±0.53 | 0.18±0.31 | 0.01±0.56 | 0.02±0.68 | 0.16±0.58 | 0.42±0.59 | -0.11±0.66 | 0.38±0.57 | 0.40±0.64 |
|  | MAE | 0.26±0.07 | 0.26±0.05 | 0.27±0.13 | 0.26±0.06 | 0.26±0.08 | 0.20±0.05 | 0.20±0.05 | 0.23±0.09 | 0.24±0.06 | 0.22±0.07 |
| 6 | RMSE | 0.32±0.09 | 0.32±0.06 | 0.30±0.13 | 0.30±0.08 | 0.34±0.08 | 0.22±0.06 | 0.25±0.08 | 0.27±0.08 | 0.29±0.08 | 0.28±0.07**^a^** |
|  | $r$ | 0.26±0.48 | -0.05±0.52 | 0.15±0.36 | 0.13±0.54 | -0.30±0.66 | 0.50±0.38 | 0.34±0.72 | 0.02±0.71 | 0.44±0.61 | 0.38±0.61 |
|  | MAE | 0.24±0.08 | 0.26±0.06 | 0.26±0.13 | 0.25±0.06 | 0.29±0.09 | 0.18±0.05 | 0.20±0.08 | 0.21±0.08 | 0.23±0.06 | 0.22±0.07 |
| 7 | RMSE | 0.31±0.10 | 0.32±0.07 | 0.30±0.13 | 0.30±0.09 | 0.34±0.09 | 0.22±0.07 | 0.25±0.07 | 0.29±0.10 | 0.27±0.08**^a^** | 0.26±0.08**^a^** |
|  | $r$ | 0.37±0.49 | -0.04±0.51 | 0.12±0.33 | 0.21±0.55 | -0.14±0.68 | 0.41±0.46 | 0.41±0.64 | -0.15±0.75 | 0.54±0.55 | 0.51±0.60 |
|  | MAE | 0.24±0.09 | 0.27±0.07 | 0.26±0.13 | 0.25±0.07 | 0.28±0.10 | 0.19±0.05 | 0.19±0.05 | 0.24±0.09 | 0.22±0.07 | 0.21±0.07 |

Note: Superscripts a, b, c, d, and e indicate significant differences compared to the number of synergies 2, 3, 4, 5, and 6, respectively. Red represents RMSE; blue represents r; purple represents MAE.

Table 3 The errors between the muscle activation predicted by the FA synergy-assisted EMG-driven NMSK model and the experimental measurements different numbers of synergies were evaluated using RMSE, $r$, and MAE.

| Number of synergies |  | Muscle | | | | | | | | | |
| --- | --- | --- | --- | --- | --- | --- | --- | --- | --- | --- | --- |
|  |  | AD | BB | BRD | IF | LD | MD | PD | PM | TB | TMj |
| 2 | RMSE | 0.25±0.14 | 0.32±0.11 | 0.37±0.09 | 0.32±0.12 | 0.43±0.20 | 0.19±0.13 | 0.16±0.09 | 0.37±0.20 | 0.18±0.10 | 0.12±0.08 |
|  | $r$ | 0.57±0.54 | 0.20±0.49 | 0.02±0.37 | 0.15±0.70 | -0.53±0.68 | 0.78±0.37 | 0.74±0.50 | 0.22±0.75 | 0.89±0.34 | 0.92±0.15 |
|  | MAE | 0.20±0.11 | 0.27±0.10 | 0.33±0.09 | 0.27±0.11 | 0.38±0.20 | 0.16±0.11 | 0.12±0.07 | 0.31±0.20 | 0.13±0.06 | 0.09±0.05 |
| 3 | RMSE | 0.27±0.17 | 0.38±0.18 | 0.36±0.07 | 0.32±0.12 | 0.52±0.22 | 0.17±0.10 | 0.18±0.10 | 0.44±0.20 | 0.18±0.09 | 0.17±0.13 |
|  | $r$ | 0.59±0.45 | 0.18±0.60 | 0.12±0.37 | 0.09±0.64 | -0.36±0.60 | 0.81±0.27 | 0.72±0.45 | -0.02±0.67 | 0.90±0.13 | 0.84±0.34 |
|  | MAE | 0.21±0.12 | 0.32±0.16 | 0.32±0.07 | 0.27±0.11 | 0.44±0.18 | 0.14±0.08 | 0.14±0.09 | 0.37±0.19 | 0.13±0.06 | 0.12±0.08 |
| 4 | RMSE | 0.24±0.11 | 0.38±0.21 | 0.36±0.07 | 0.32±0.13 | 0.47±0.20 | 0.19±0.12 | 0.17±0.09 | 0.38±0.17 | 0.19±0.07 | 0.16±0.16 |
|  | $r$ | 0.62±0.37 | 0.16±0.50 | 0.13±0.37 | 0.15±0.59 | -0.29±0.65 | 0.78±0.29 | 0.72±0.39 | 0.13±0.61 | 0.86±0.26 | 0.83±0.33 |
|  | MAE | 0.19±0.09 | 0.31±0.17 | 0.31±0.08 | 0.27±0.12 | 0.39±0.18 | 0.16±0.10 | 0.13±0.07 | 0.31±0.15 | 0.14±0.05 | 0.12±0.13 |
| 5 | RMSE | 0.32±0.23 | 0.36±0.21 | 0.36±0.06 | 0.34±0.11 | 0.54±0.29 | 0.16±0.07 | 0.17±0.07 | 0.46±0.24 | 0.19±0.10 | 0.15±0.07 |
|  | $r$ | 0.51±0.49 | 0.25±0.50 | 0.11±0.36 | 0.14±0.54 | -0.35±0.49 | 0.82±0.18 | 0.71±0.38 | 0.04±0.61 | 0.82±0.29 | 0.89±0.12 |
|  | MAE | 0.26±0.19 | 0.30±0.18 | 0.31±0.07 | 0.28±0.11 | 0.45±0.23 | 0.13±0.06 | 0.12±0.05 | 0.38±0.21 | 0.14±0.07 | 0.10±0.04 |
| 6 | RMSE | 0.30±0.20 | 0.33±0.13 | 0.36±0.06 | 0.39±0.23 | 0.56±0.22 | 0.17±0.08 | 0.17±0.07 | 0.46±0.31 | 0.19±0.08 | 0.14±0.04 |
|  | $r$ | 0.58±0.39 | 0.21±0.46 | 0.10±0.36 | 0.14±0.52 | -0.54±0.45 | 0.82±0.15 | 0.66±0.39 | 0.00±0.65 | 0.81±0.33 | 0.91±0.05**^a^** |
|  | MAE | 0.24±0.17 | 0.27±0.11 | 0.31±0.06 | 0.32±0.17 | 0.49±0.19 | 0.14±0.07 | 0.12±0.05 | 0.37±0.24 | 0.14±0.06 | 0.10±0.03 |
| 7 | RMSE | 0.32±0.24 | 0.51±0.33 | 0.37±0.05 | 0.36±0.11 | 0.48±0.16 | 0.18±0.09 | 0.18±0.09 | 0.47±0.21 | 0.20±0.18 | 0.15±0.06 |
|  | $r$ | 0.57±0.43 | 0.12±0.42 | 0.10±0.38 | 0.16±0.48 | -0.30±0.58 | 0.80±0.19 | 0.64±0.44 | -0.05±0.55 | 0.83±0.29 | 0.89±0.07**^a^** |
|  | MAE | 0.26±0.20 | 0.42±0.26 | 0.31±0.05 | 0.30±0.11 | 0.40±0.14 | 0.14±0.08 | 0.13±0.06 | 0.39±0.18 | 0.15±0.14 | 0.11±0.04 |

Note: Superscripts a, b, c, d, and e indicate significant differences compared to the number of synergies 2, 3, 4, 5, and 6, respectively. Red represents RMSE; blue represents r; purple represents MAE.

Table 4 The errors between the muscle activation predicted by the NMF synergy-assisted EMG-driven NMSK model and the experimental measurements different numbers of synergies were evaluated using RMSE, $r$, and MAE.

| Number of  synergies |  | Muscle | | | | | | | | | |
| --- | --- | --- | --- | --- | --- | --- | --- | --- | --- | --- | --- |
|  |  | AD | BB | BRD | IF | LD | MD | PD | PM | TB | TMj |
| 2 | RMSE | 0.27±0.16 | 0.28±0.13 | 0.35±0.15 | 0.28±0.14 | 0.34±0.07 | 0.14±0.04 | 0.14±0.07 | 0.24±0.07 | 0.16±0.07 | 0.10±0.03 |
|  | $r$ | 0.83±0.25 | 0.69±0.30 | 0.44±0.32 | 0.84±0.14 | 0.94±0.06 | 0.93±0.04 | 0.94±0.04 | 0.90±0.05 | 0.96±0.03 | 0.96±0.02 |
|  | MAE | 0.23±0.14 | 0.23±0.11 | 0.31±0.15 | 0.24±0.13 | 0.25±0.05 | 0.11±0.04 | 0.11±0.05 | 0.16±0.04 | 0.12±0.05 | 0.07±0.02 |
| 3 | RMSE | 0.26±0.13 | 0.27±0.10 | 0.31±0.16 | 0.27±0.13 | 0.32±0.07 | 0.14±0.04 | 0.14±0.07 | 0.23±0.07 | 0.17±0.07 | 0.11±0.03 |
|  | $r$ | 0.80±0.32 | 0.58±0.33 | 0.41±0.36 | 0.76±0.21 | 0.91±0.09 | 0.91±0.04 | 0.93±0.07 | 0.84±0.17 | 0.92±0.06 | 0.95±0.03 |
|  | MAE | 0.21±0.11 | 0.22±0.09 | 0.28±0.16 | 0.23±0.12 | 0.24±0.05 | 0.12±0.04 | 0.11±0.05 | 0.15±0.05 | 0.13±0.05 | 0.08±0.03 |
| 4 | RMSE | 0.23±0.13 | 0.26±0.11 | 0.30±0.14 | 0.25±0.10 | 0.30±0.09 | 0.14±0.04 | 0.15±0.07 | 0.22±0.07 | 0.18±0.10 | 0.12±0.03 |
|  | $r$ | 0.84±0.14 | 0.59±0.32 | 0.37±0.39 | 0.77±0.14 | 0.81±0.25**^a^** | 0.89±0.06 | 0.89±0.11 | 0.78±0.24 | 0.94±0.06 | 0.94±0.04 |
|  | MAE | 0.19±0.11 | 0.21±0.09 | 0.26±0.14 | 0.21±0.09 | 0.22±0.07 | 0.12±0.03 | 0.11±0.05 | 0.15±0.06 | 0.14±0.08 | 0.09±0.02 |
| 5 | RMSE | 0.21±0.10 | 0.26±0.12 | 0.29±0.13 | 0.25±0.09 | 0.27±0.12**^a^** | 0.15±0.04 | 0.14±0.06 | 0.20±0.06 | 0.17±0.10 | 0.15±0.12**^a^** |
|  | $r$ | 0.80±0.18 | 0.62±0.28 | 0.38±0.36 | 0.76±0.15**^a^** | 0.81±0.26**^a^** | 0.91±0.05 | 0.88±0.11 | 0.84±0.10 | 0.94±0.06 | 0.93±0.06 |
|  | MAE | 0.17±0.08 | 0.21±0.09 | 0.25±0.13 | 0.21±0.09 | 0.20±0.09**^a^** | 0.12±0.03 | 0.11±0.04 | 0.14±0.03 | 0.14±0.09 | 0.12±0.11**^a^** |
| 6 | RMSE | 0.19±0.10 | 0.22±0.09 | 0.32±0.16 | 0.25±0.12 | 0.27±0.15**^ab^** | 0.14±0.04 | 0.16±0.10 | 0.24±0.16 | 0.16±0.07 | 0.13±0.04**^a^** |
|  | $r$ | 0.84±0.12 | 0.64±0.28 | 0.27±0.28 | 0.78±0.19 | 0.82±0.29**^a^** | 0.89±0.06**^a^** | 0.89±0.09 | 0.82±0.11 | 0.92±0.06 | 0.91±0.14**^a^** |
|  | MAE | 0.16±0.08 | 0.17±0.07 | 0.27±0.14 | 0.20±0.11 | 0.21±0.14**^a^** | 0.12±0.03 | 0.13±0.09 | 0.18±0.15 | 0.13±0.05 | 0.10±0.04**^a^** |
| 7 | RMSE | 0.23±0.12 | 0.31±0.19 | 0.29±0.18 | 0.31±0.19 | 0.22±0.12**^abc^** | 0.15±0.08 | 0.17±0.13 | 0.25±0.16 | 0.17±0.06 | 0.16±0.13**^a^** |
|  | $r$ | 0.85±0.11 | 0.63±0.29 | 0.28±0.40 | 0.80±0.12 | 0.87±0.12**^a^** | 0.89±0.07 | 0.89±0.14 | 0.79±0.19 | 0.92±0.07**^a^** | 0.91±0.10**^a^** |
|  | MAE | 0.19±0.11 | 0.25±0.17 | 0.24±0.15 | 0.25±0.16 | 0.17±0.11**^abc^** | 0.13±0.07 | 0.13±0.11 | 0.20±0.15 | 0.14±0.04 | 0.13±0.12**^a^** |

Note: Superscripts a, b, c, d, and e indicate significant differences compared to the number of synergies 2, 3, 4, 5, and 6, respectively. Red represents RMSE; blue represents r; purple represents MAE.

Table 5 RMSE and $r$ between the motor primitive curves of the known muscle activation matrix (with one missing muscle) and the full 10-muscle activation matrix derived from NMF decomposition.

| Synergy number | Motor primitives | |
| --- | --- | --- |
|  | RMSE | $r$ |
| 2 | 0.090 (±0.056) | 0.965 (±0.083) |
| 3 | 0.106 (±0.084) | 0.894 (±0.211)^a^ |
| 4 | 0.112 (±0.074)^a^ | 0.852 (±0.237)^ab^ |
| 5 | 0.103 (±0.062) | 0.833 (±0.268)^ab^ |
| 6 | 0.123 (±0.069)^abcd^ | 0.725 (±0.333)^abcd^ |
| 7 | 0.112 (±0.062)^abd^ | 0.717 (±0.317)^abcd^ |

Note: Superscripts a, b, c, d, and e indicate significant differences compared to the number of synergies 2, 3, 4, 5, and 6, respectively.

Table 6 The %RMSE, $r$ and %MAE values of the arm adduction-abduction moments predicted by the NMF synergy-assisted EMG-driven NMSK muscle force estimation model from varying numbers of synergies, compared to the experimentally measured moments.

| **Number of synergies** |  | **Missing muscle** | | | | | | | | | |
| --- | --- | --- | --- | --- | --- | --- | --- | --- | --- | --- | --- |
|  |  | **AD** | **BB** | **BRD** | **IF** | **LD** | **MD** | **PD** | **PM** | **TB** | **TMj** |
| 2 | %RMSE | 0.34±0.16 | 0.34±0.16 | 0.34±0.16 | 0.35±0.13 | 0.33±0.14 | 0.32±0.16 | 0.26±0.09 | 0.34±0.15 | 0.25±0.10 | 0.32±0.15 |
|  | $r$ | 0.80±0.17 | 0.80±0.17 | 0.80±0.17 | 0.82±0.15 | 0.79±0.17 | 0.82±0.16 | 0.82±0.12 | 0.80±0.17 | 0.78±0.18 | 0.82±0.16 |
|  | %MAE | 0.25±0.12 | 0.25±0.12 | 0.26±0.12 | 0.25±0.09 | 0.25±0.12 | 0.25±0.13 | 0.20±0.08 | 0.25±0.12 | 0.19±0.08 | 0.25±0.13 |
| 3 | %RMSE | 0.34±0.16 | 0.34±0.16 | 0.34±0.16 | 0.34±0.13 | 0.33±0.15 | 0.30±0.15 | 0.25±0.09 | 0.34±0.16 | 0.23±0.09 | 0.31±0.14 |
|  | $r$ | 0.80±0.16 | 0.80±0.17 | 0.80±0.17 | 0.84±0.12 | 0.79±0.17 | 0.86±0.11 | 0.84±0.10 | 0.80±0.17 | 0.82±0.13 | 0.84±0.13 |
|  | %MAE | 0.25±0.12 | 0.25±0.12 | 0.26±0.12 | 0.25±0.09 | 0.25±0.12 | 0.23±0.11 | 0.19±0.08 | 0.25±0.12 | 0.18±0.07 | 0.25±0.13 |
| 4 | %RMSE | 0.34±0.16 | 0.34±0.16 | 0.34±0.16 | 0.33±0.13 | 0.33±0.15 | 0.30±0.15 | 0.24±0.10 | 0.34±0.16 | 0.24±0.09 | 0.31±0.14 |
|  | $r$ | 0.80±0.17 | 0.80±0.17 | 0.80±0.17 | 0.83±0.13 | 0.79±0.17 | 0.85±0.14 | 0.85±0.08 | 0.80±0.17 | 0.82±0.13 | 0.84±0.14 |
|  | %MAE | 0.25±0.12 | 0.25±0.12 | 0.26±0.12 | 0.24±0.09 | 0.25±0.12 | 0.23±0.12 | 0.19±0.08 | 0.25±0.12 | 0.19±0.08 | 0.24±0.12 |
| 5 | %RMSE | 0.34±0.16 | 0.34±0.16 | 0.34±0.16 | 0.33±0.13 | 0.33±0.15 | 0.30±0.13 | 0.25±0.11 | 0.34±0.16 | 0.25±0.10 | 0.32±0.16 |
|  | $r$ | 0.80±0.17 | 0.80±0.17 | 0.80±0.17 | 0.84±0.11 | 0.80±0.17 | 0.83±0.17 | 0.84±0.11 | 0.80±0.17 | 0.82±0.14 | 0.86±0.14 |
|  | %MAE | 0.25±0.12 | 0.25±0.12 | 0.26±0.12 | 0.24±0.09 | 0.25±0.12 | 0.23±0.10 | 0.20±0.08 | 0.25±0.12 | 0.19±0.09 | 0.25±0.13 |
| 6 | %RMSE | 0.34±0.16 | 0.34±0.16 | 0.34±0.16 | 0.34±0.13 | 0.34±0.16 | 0.29±0.13 | 0.29±0.14 | 0.34±0.16 | 0.25±0.10 | 0.31±0.15 |
|  | $r$ | 0.80±0.17 | 0.80±0.17 | 0.80±0.17 | 0.83±0.13 | 0.80±0.17 | 0.85±0.11 | 0.84±0.11 | 0.80±0.17 | 0.83±0.12 | 0.85±0.14 |
|  | %MAE | 0.25±0.12 | 0.25±0.12 | 0.26±0.12 | 0.25±0.10 | 0.25±0.12 | 0.23±0.10 | 0.22±0.10 | 0.25±0.12 | 0.19±0.08 | 0.24±0.13 |
| 7 | %RMSE | 0.34±0.16 | 0.34±0.16 | 0.34±0.16 | 0.33±0.13 | 0.34±0.15 | 0.32±0.17 | 0.31±0.17 | 0.34±0.16 | 0.24±0.12 | 0.33±0.19 |
|  | $r$ | 0.80±0.17 | 0.80±0.17 | 0.80±0.17 | 0.83±0.11 | 0.80±0.17 | 0.80±0.25 | 0.84±0.11 | 0.80±0.17 | 0.81±0.18 | 0.85±0.12 |
|  | %MAE | 0.26±0.12 | 0.25±0.12 | 0.26±0.12 | 0.24±0.09 | 0.25±0.12 | 0.24±0.12 | 0.24±0.14 | 0.25±0.12 | 0.18±0.09 | 0.26±0.15 |

Note: Gray shading indicates a significant difference from other conditions ($p$< 0.05).

Table 7 The %RMSE, $r$ and %MAE values of the arm flexion-extension moments predicted by the NMF synergy-assisted EMG-driven NMSK muscle force estimation model from varying numbers of synergies, compared to the experimentally measured moments.

| **Number of synergies** |  | **Missing muscle** | | | | | | | | | |
| --- | --- | --- | --- | --- | --- | --- | --- | --- | --- | --- | --- |
|  |  | **AD** | **BB** | **BRD** | **IF** | **LD** | **MD** | **PD** | **PM** | **TB** | **TMj** |
| 2 | %RMSE | 0.17±0.03 | 0.17±0.03 | 0.17±0.03 | 0.17±0.04 | 0.17±0.03 | 0.17±0.03 | 0.20±0.06 | 0.17±0.03 | 0.18±0.04 | 0.17±0.04 |
|  | $r$ | 0.91±0.05 | 0.91±0.06 | 0.91±0.06 | 0.91±0.06 | 0.91±0.06 | 0.90±0.05 | 0.89±0.07 | 0.91±0.06 | 0.89±0.06 | 0.91±0.06 |
|  | %MAE | 0.14±0.02 | 0.14±0.03 | 0.14±0.03 | 0.14±0.03 | 0.14±0.03 | 0.14±0.03 | 0.17±0.05 | 0.14±0.03 | 0.15±0.03 | 0.14±0.03 |
| 3 | %RMSE | 0.17±0.03 | 0.17±0.03 | 0.17±0.03 | 0.16±0.04 | 0.17±0.03 | 0.17±0.03 | 0.20±0.06 | 0.17±0.03 | 0.18±0.04 | 0.17±0.04 |
|  | $r$ | 0.91±0.05 | 0.91±0.06 | 0.91±0.06 | 0.91±0.06 | 0.91±0.06 | 0.90±0.06 | 0.90±0.05 | 0.91±0.06 | 0.89±0.06 | 0.91±0.06 |
|  | %MAE | 0.14±0.02 | 0.14±0.03 | 0.14±0.03 | 0.14±0.03 | 0.14±0.03 | 0.14±0.03 | 0.17±0.04 | 0.14±0.03 | 0.15±0.03 | 0.14±0.03 |
| 4 | %RMSE | 0.17±0.03 | 0.17±0.03 | 0.17±0.03 | 0.17±0.04 | 0.17±0.03 | 0.18±0.03 | 0.20±0.06 | 0.17±0.03 | 0.18±0.04 | 0.17±0.04 |
|  | $r$ | 0.91±0.06 | 0.91±0.06 | 0.91±0.06 | 0.91±0.05 | 0.91±0.06 | 0.90±0.06 | 0.91±0.05 | 0.91±0.06 | 0.90±0.06 | 0.91±0.06 |
|  | %MAE | 0.14±0.02 | 0.14±0.03 | 0.14±0.03 | 0.14±0.03 | 0.14±0.03 | 0.15±0.03 | 0.16±0.05 | 0.14±0.03 | 0.15±0.03 | 0.14±0.03 |
| 5 | %RMSE | 0.17±0.03 | 0.17±0.03 | 0.17±0.03 | 0.17±0.04 | 0.17±0.03 | 0.18±0.03 | 0.20±0.06 | 0.17±0.03 | 0.18±0.04 | 0.17±0.04 |
|  | $r$ | 0.91±0.06 | 0.91±0.06 | 0.91±0.06 | 0.91±0.05 | 0.91±0.06 | 0.90±0.06 | 0.89±0.08 | 0.91±0.06 | 0.90±0.06 | 0.91±0.06 |
|  | %MAE | 0.14±0.02 | 0.14±0.03 | 0.14±0.03 | 0.14±0.03 | 0.14±0.03 | 0.15±0.03 | 0.17±0.04 | 0.14±0.03 | 0.15±0.03 | 0.15±0.03 |
| 6 | %RMSE | 0.17±0.03 | 0.17±0.03 | 0.17±0.03 | 0.17±0.04 | 0.17±0.03 | 0.18±0.04 | 0.20±0.06 | 0.17±0.03 | 0.18±0.04 | 0.17±0.03 |
|  | $r$ | 0.91±0.06 | 0.91±0.06 | 0.91±0.06 | 0.91±0.06 | 0.91±0.06 | 0.90±0.06 | 0.88±0.07 | 0.91±0.06 | 0.90±0.06 | 0.91±0.05 |
|  | %MAE | 0.14±0.02 | 0.14±0.03 | 0.14±0.03 | 0.14±0.03 | 0.14±0.03 | 0.15±0.03 | 0.17±0.05 | 0.14±0.03 | 0.15±0.03 | 0.15±0.03 |
| 7 | %RMSE | 0.17±0.03 | 0.17±0.03 | 0.17±0.03 | 0.17±0.04 | 0.17±0.03 | 0.18±0.04 | 0.21±0.07 | 0.17±0.03 | 0.18±0.04 | 0.18±0.04 |
|  | $r$ | 0.91±0.06 | 0.91±0.06 | 0.91±0.06 | 0.91±0.05 | 0.91±0.06 | 0.89±0.06 | 0.88±0.08 | 0.91±0.06 | 0.90±0.06 | 0.90±0.07 |
|  | %MAE | 0.14±0.02 | 0.14±0.03 | 0.14±0.03 | 0.14±0.03 | 0.14±0.03 | 0.15±0.03 | 0.17±0.05 | 0.14±0.03 | 0.15±0.03 | 0.15±0.03 |

Note: Gray shading indicates a significant difference from other conditions ($p$< 0.05).

Table 8 The %RMSE, $r$ and %MAE values of the arm internal-external rotation moments predicted by the NMF synergy-assisted EMG-driven NMSK muscle force estimation model from varying numbers of synergies, compared to the experimentally measured moments.

| **Number of synergies** |  | **Missing muscle** | | | | | | | | | |
| --- | --- | --- | --- | --- | --- | --- | --- | --- | --- | --- | --- |
|  |  | **AD** | **BB** | **BRD** | **IF** | **LD** | **MD** | **PD** | **PM** | **TB** | **TMj** |
| 2 | %RMSE | 0.35±0.11 | 0.35±0.11 | 0.35±0.11 | 0.29±0.06 | 0.34±0.12 | 0.35±0.11 | 0.35±0.11 | 0.35±0.11 | 0.35±0.11 | 0.35±0.10 |
|  | $r$ | 0.54±0.26 | 0.54±0.26 | 0.54±0.26 | 0.67±0.18 | 0.58±0.27 | 0.57±0.28 | 0.54±0.25 | 0.55±0.26 | 0.56±0.25 | 0.47±0.24 |
|  | %MAE | 0.27±0.08 | 0.27±0.08 | 0.27±0.08 | 0.22±0.04 | 0.25±0.09 | 0.26±0.08 | 0.26±0.08 | 0.26±0.08 | 0.27±0.08 | 0.27±0.07 |
| 3 | %RMSE | 0.35±0.11 | 0.35±0.11 | 0.35±0.11 | 0.29±0.06 | 0.34±0.12 | 0.34±0.11 | 0.35±0.11 | 0.35±0.11 | 0.35±0.11 | 0.35±0.10 |
|  | $r$ | 0.55±0.26 | 0.54±0.26 | 0.54±0.26 | 0.69±0.15 | 0.58±0.26 | 0.57±0.26 | 0.54±0.25 | 0.55±0.26 | 0.55±0.25 | 0.50±0.23 |
|  | %MAE | 0.27±0.08 | 0.27±0.08 | 0.27±0.08 | 0.22±0.04 | 0.26±0.09 | 0.26±0.08 | 0.26±0.08 | 0.26±0.08 | 0.27±0.08 | 0.27±0.07 |
| 4 | %RMSE | 0.35±0.11 | 0.35±0.11 | 0.35±0.11 | 0.30±0.06 | 0.34±0.12 | 0.34±0.11 | 0.35±0.11 | 0.35±0.11 | 0.35±0.11 | 0.35±0.10 |
|  | $r$ | 0.54±0.26 | 0.54±0.26 | 0.54±0.26 | 0.66±0.16 | 0.57±0.26 | 0.57±0.27 | 0.54±0.24 | 0.55±0.26 | 0.55±0.25 | 0.50±0.23 |
|  | %MAE | 0.27±0.08 | 0.27±0.08 | 0.27±0.08 | 0.23±0.04 | 0.26±0.09 | 0.26±0.08 | 0.27±0.08 | 0.26±0.08 | 0.27±0.08 | 0.27±0.07 |
| 5 | %RMSE | 0.35±0.11 | 0.35±0.11 | 0.35±0.11 | 0.30±0.07 | 0.34±0.12 | 0.34±0.11 | 0.35±0.11 | 0.35±0.11 | 0.35±0.11 | 0.34±0.10 |
|  | $r$ | 0.55±0.26 | 0.54±0.26 | 0.54±0.26 | 0.66±0.18 | 0.57±0.26 | 0.57±0.26 | 0.54±0.25 | 0.55±0.26 | 0.55±0.25 | 0.52±0.23 |
|  | %MAE | 0.27±0.08 | 0.27±0.08 | 0.27±0.08 | 0.23±0.04 | 0.26±0.09 | 0.25±0.08 | 0.27±0.08 | 0.26±0.08 | 0.27±0.08 | 0.26±0.07 |
| 6 | %RMSE | 0.35±0.11 | 0.35±0.11 | 0.35±0.11 | 0.33±0.14 | 0.35±0.12 | 0.34±0.11 | 0.35±0.11 | 0.35±0.11 | 0.35±0.11 | 0.35±0.11 |
|  | $r$ | 0.55±0.26 | 0.54±0.26 | 0.54±0.26 | 0.60±0.31 | 0.55±0.26 | 0.57±0.26 | 0.54±0.24 | 0.55±0.26 | 0.55±0.25 | 0.50±0.30 |
|  | %MAE | 0.27±0.08 | 0.27±0.08 | 0.27±0.08 | 0.26±0.11 | 0.26±0.09 | 0.25±0.08 | 0.27±0.08 | 0.26±0.08 | 0.27±0.08 | 0.27±0.07 |
| 7 | %RMSE | 0.35±0.11 | 0.35±0.11 | 0.35±0.11 | 0.33±0.11 | 0.35±0.12 | 0.35±0.11 | 0.35±0.11 | 0.35±0.11 | 0.35±0.11 | 0.34±0.10 |
|  | $r$ | 0.55±0.26 | 0.54±0.26 | 0.54±0.26 | 0.58±0.22 | 0.56±0.26 | 0.56±0.25 | 0.55±0.25 | 0.55±0.26 | 0.55±0.25 | 0.51±0.28 |
|  | %MAE | 0.27±0.08 | 0.27±0.08 | 0.27±0.08 | 0.25±0.09 | 0.26±0.09 | 0.26±0.08 | 0.26±0.08 | 0.26±0.08 | 0.27±0.08 | 0.26±0.06 |

Note: Gray shading indicates a significant difference from other conditions ($p$< 0.05).

Table 9 The %RMSE, $r$ and %MAE values of the elbow flexion-extension moments predicted by the NMF synergy-assisted EMG-driven NMSK muscle force estimation model from varying numbers of synergies, compared to the experimentally measured moments.

| **Number of synergies** |  | **Missing muscle** | | | | | | | | | |
| --- | --- | --- | --- | --- | --- | --- | --- | --- | --- | --- | --- |
|  |  | **AD** | **BB** | **BRD** | **IF** | **LD** | **MD** | **PD** | **PM** | **TB** | **TMj** |
| 2 | %RMSE | 0.17±0.03 | 0.17±0.03 | 0.16±0.03 | 0.17±0.03 | 0.17±0.03 | 0.17±0.03 | 0.17±0.03 | 0.17±0.03 | 0.23±0.06 | 0.17±0.03 |
|  | $r$ | 0.91±0.07 | 0.91±0.07 | 0.91±0.06 | 0.91±0.07 | 0.91±0.07 | 0.91±0.07 | 0.91±0.07 | 0.91±0.07 | 0.90±0.06 | 0.91±0.07 |
|  | %MAE | 0.14±0.02 | 0.14±0.02 | 0.14±0.02 | 0.14±0.02 | 0.14±0.02 | 0.14±0.02 | 0.14±0.02 | 0.14±0.02 | 0.19±0.04 | 0.14±0.02 |
| 3 | %RMSE | 0.17±0.03 | 0.17±0.03 | 0.16±0.03 | 0.17±0.03 | 0.17±0.03 | 0.17±0.03 | 0.17±0.03 | 0.17±0.03 | 0.23±0.06 | 0.17±0.03 |
|  | $r$ | 0.91±0.07 | 0.91±0.07 | 0.92±0.06 | 0.91±0.07 | 0.91±0.07 | 0.91±0.07 | 0.91±0.07 | 0.91±0.07 | 0.90±0.06 | 0.91±0.07 |
|  | %MAE | 0.14±0.02 | 0.14±0.02 | 0.14±0.02 | 0.14±0.02 | 0.14±0.02 | 0.14±0.02 | 0.14±0.02 | 0.14±0.02 | 0.19±0.04 | 0.14±0.02 |
| 4 | %RMSE | 0.17±0.03 | 0.17±0.03 | 0.16±0.03 | 0.17±0.03 | 0.17±0.03 | 0.17±0.03 | 0.17±0.03 | 0.17±0.03 | 0.23±0.05 | 0.17±0.03 |
|  | $r$ | 0.91±0.07 | 0.91±0.07 | 0.92±0.06 | 0.91±0.07 | 0.91±0.07 | 0.91±0.07 | 0.91±0.07 | 0.91±0.07 | 0.90±0.05 | 0.91±0.07 |
|  | %MAE | 0.14±0.02 | 0.14±0.02 | 0.14±0.02 | 0.14±0.02 | 0.14±0.02 | 0.14±0.02 | 0.14±0.02 | 0.14±0.02 | 0.19±0.04 | 0.14±0.02 |
| 5 | %RMSE | 0.17±0.03 | 0.17±0.03 | 0.16±0.03 | 0.17±0.03 | 0.17±0.03 | 0.17±0.03 | 0.17±0.03 | 0.17±0.03 | 0.23±0.06 | 0.17±0.03 |
|  | $r$ | 0.91±0.07 | 0.91±0.07 | 0.92±0.06 | 0.91±0.07 | 0.91±0.07 | 0.91±0.07 | 0.91±0.07 | 0.91±0.07 | 0.89±0.08 | 0.91±0.07 |
|  | %MAE | 0.14±0.02 | 0.14±0.02 | 0.14±0.02 | 0.14±0.02 | 0.14±0.02 | 0.14±0.02 | 0.14±0.02 | 0.14±0.02 | 0.19±0.05 | 0.14±0.02 |
| 6 | %RMSE | 0.17±0.03 | 0.17±0.03 | 0.18±0.04 | 0.17±0.03 | 0.17±0.03 | 0.17±0.03 | 0.17±0.03 | 0.17±0.03 | 0.22±0.05 | 0.17±0.03 |
|  | $r$ | 0.91±0.07 | 0.91±0.07 | 0.90±0.08 | 0.91±0.07 | 0.91±0.07 | 0.91±0.07 | 0.91±0.07 | 0.91±0.07 | 0.89±0.09 | 0.91±0.07 |
|  | %MAE | 0.14±0.02 | 0.14±0.02 | 0.15±0.03 | 0.14±0.02 | 0.14±0.02 | 0.14±0.02 | 0.14±0.02 | 0.14±0.02 | 0.18±0.04 | 0.14±0.02 |
| 7 | %RMSE | 0.17±0.03 | 0.18±0.03 | 0.18±0.04 | 0.17±0.03 | 0.17±0.03 | 0.17±0.03 | 0.17±0.03 | 0.17±0.03 | 0.23±0.05 | 0.17±0.03 |
|  | $r$ | 0.91±0.07 | 0.91±0.07 | 0.91±0.06 | 0.91±0.07 | 0.91±0.07 | 0.91±0.07 | 0.91±0.07 | 0.91±0.07 | 0.88±0.10 | 0.91±0.07 |
|  | %MAE | 0.14±0.02 | 0.14±0.02 | 0.15±0.03 | 0.14±0.02 | 0.14±0.02 | 0.14±0.02 | 0.14±0.02 | 0.14±0.02 | 0.19±0.04 | 0.14±0.02 |

Note: Gray shading indicates a significant difference from other conditions ($p$< 0.05).
